# Synchronisation of circadian timing in families and the impact of autism: a scoping review

**DOI:** 10.1186/s11689-026-09679-z

**Published:** 2026-02-25

**Authors:** Aoife Brennan, Cathy Wyse, Mailton Vasconcelos, Laura Rudderham, Louise Gallagher, Lorna M. Lopez

**Affiliations:** 1 Department of Biology, Maynooth University, Maynooth, Co. Kildare Ireland; 2 FutureNeuro Research Ireland Centre, Department of Biology, Maynooth University, Co. Kildare Maynooth, Ireland; 3 The Hospital for SickKids, the Centre for Addiction and Mental Health and the University of Toronto, Toronto, Canada; 4 School of Medicine, Trinity College Dublin, Co. Dublin Dublin, Ireland

**Keywords:** Autism, Sleep Synchrony, Family, Circadian Rhythm Disruption, Scoping Review

## Abstract

Autism Spectrum Disorder (autism) is a neurodevelopmental condition characterised by altered social communication, repetitive behaviours and restricted interests. Emerging evidence suggests that disrupted circadian timing mechanisms may be associated with certain traits of autism, such as sleep difficulties and behavioural dysregulation. Circadian rhythms are primarily entrained by the light–dark cycle but are also shaped by social cues, including synchronisation with the circadian rhythms of family members. Despite growing interest in circadian biology and autism, little is known about how circadian synchrony functions within families that include autistic children. This scoping review synthesises findings on circadian timing in families with autistic and non-autistic children. A systematic search of four databases (PubMed, Scopus, PsycINFO, and CINAHL) yielded 2,423 results, of which 25 met inclusion criteria. An additional 17 studies were identified through handsearching. The final dataset comprised 42 studies, which were organised into a narrative synthesis and thematic analysis. Six studies focused on sleep synchrony in families with autistic children, while the remaining 36 studies examined circadian biomarkers (*n* = 5), activity rhythms (*n* = 12), and sleep synchrony (*n* = 19) in families with non-autistic children. Findings revealed evidence of circadian synchrony in both groups, with family members displaying aligned sleep–wake patterns and daily activity rhythms. However, studies involving autistic families were relatively limited, and the mechanisms underlying circadian synchrony remain poorly understood. While this review highlights emerging insights into circadian synchrony in families, significant gaps in the literature remain. Research in autistic families is particularly scarce, and there is a need for longitudinal and experimental studies to better understand the interplay between genetic, environmental and social factors. Further research should explore whether disrupted circadian synchrony contributes to the neurobiology of autism or its associated traits, such as sleep difficulties and behavioural dysregulation. Addressing these gaps could inform targeted interventions to improve sleep and overall well-being in autistic children and their families.

**Supplementary Information:**

The online version contains supplementary material available at 10.1186/s11689-026-09679-z.

## Background

Autism Spectrum Disorder (herein referred to as autism) is one of the most common neurodevelopmental conditions, with an estimated worldwide prevalence of approximately 0.6% (with a range of 0.02% in China-3.66% in Sweden) [1]. Autism is an early onset condition, characterised by altered social communication, repetitive behaviours and restricted interests [2]. The heritability of autism is high [3, 4], with research on the genetic basis of the condition implicating rare and common inherited variation, as well as acquired de novo mutations [5–8]. However, both genetic and environmental factors contribute to the aetiology of autism.

Autism is a highly heterogeneous condition, encompassing a wide range of cognitive profiles, adaptive functioning levels and co-occurring conditions such as intellectual disability, developmental delay and co-occurring mental health disorders. Research studies link autism to circadian timing, particularly disruption of the sleep-wake cycle and differences in the daily secretion profiles of circadian hormones such as cortisol and melatonin [9–12]. Further research suggests an association between disrupted circadian timing and specific traits of autism [13]. For example, sleep difficulties are one of the most common co-occurring traits in autistic individuals, and an association between these sleep difficulties and other features characteristic of the condition is often highlighted [14, 15]. As such, researchers propose that disrupted circadian rhythmicity may be associated with traits commonly observed in autism [16], though further research is needed to clarify its role in neurobiology . One of the suggested mechanisms for this disrupted circadian timing in autistic individuals is atypical functioning of circadian genes [17, 18], but results in this field are currently preliminary. 

Circadian rhythms are endogenous oscillations in physiology and behaviour that recur within a period of 24 h and that persist or “free run” in the absence of external time cues. Circadian rhythms are regulated by a master clock located in the hypothalamic suprachiasmatic nuclei (SCN). This master clock is synchronised daily by means of *zeitgebers* (environmental time cues). The light–dark cycle acts as the strongest *zeitgeber*. The daily photoperiod generated by the rotation of the Earth resets the phase of the endogenous circadian clock at each 24-h cycle in a process known as entrainment [19]. While the light–dark cycle is considered the dominant *zeitgeber* it is not the only environmental stimulus that can entrain circadian rhythms. Non-photic stimuli, such as food availability, feeding schedules [20–22], and social cues [23–26] also contribute to circadian entrainment.

Certain species, such as honeybees, can be entrained by exposure to conspecifics, resulting in mutual synchronisation of circadian rhythms between members of the same species [23–28], sometimes even in the presence of conflicting photic cues [24]. In humans, social cues may contribute to circadian entrainment, particularly when combined with structured routines or other stimuli [29, 30]. However, empirical evidence suggests these effects are relatively weak when isolated [31], as expected given the strong and well-established influence of light on the human circadian system.

Daily social habits and cues that are driven by behaviour rather than endogenous biological rhythms, such as mealtimes and exercise, sleep schedules, and work schedules (e.g., night-work) have also been shown to indirectly phase shift the circadian clock [32]. These social cues can sometimes be asynchronous with our innate biological timing (e.g., mistimed meals or sleep) and this can result in a discrepancy between environmental cues and the body’s internal time, which is known as social jetlag or circadian misalignment [33]. This is associated with negative effects on health and wellbeing [32, 34]. It is important to note that there are interindividual differences in biological timing in humans, particularly sleep timing. For example, some individuals have a preference to be active early in the day (morning types), while others have a preference for evening time (evening types) [35]. This is referred to as an individual’s chronotype [36]. Chronotype can change across developmental stages. For example, there is typically a shift towards an evening chronotype after puberty [37].

Nearly all physiological and behavioural functions in humans are rhythmic and humans tend to coordinate with social partners when they interact with one another, in a process known as interpersonal synchrony [38]. Interpersonal synchrony involves the temporal coordination or synchronisation of behaviour, affect and biological states [39, 40]. Interpersonal synchrony is a behavioural construct, but it is likely that the social entrainment of the circadian clock contributes to this synchronisation. Interpersonal synchrony begins shortly after birth, gradually becomes more complex and dynamic [41], and is typically greater in those with a close bond [42]. There is strong evidence that parents and children coordinate their behaviour and physiology, and that this interpersonal synchrony is critical for children’s healthy developmental outcomes [43–45].

Research on parent–child interpersonal synchrony has predominately focused on the temporal coordination of high frequency behavioural and physiological rhythms over short periods of time (less than 24 h), such as the coordination of gaze, affect, touch, body orientation and manual actions [46], skin temperature, heart rate, and cortisol levels [47–53]. However, little research has focused on the synchrony of behavioural and physiological rhythms at periods of 24 h (circadian) between cohabiting family members and how this may impact family functioning and health.

Drawing on a family systems approach [54], which posits that individuals are best understood in the context of the family to which they belong, it is reasonable to assume that the functioning or behaviour of one family member can influence the functioning or behaviour of another family member, and that this could potentially extend to circadian timing. Research shows that parent and child sleep is interrelated and that disrupted sleep timing in one family member can subsequently impact the sleep timing of other family members, thus impacting circadian timing. For example, mother’s insomnia symptoms are related to their child’s sleep patterns [55] and children’s sleep difficulties can impact parent’s sleep quality and mood [56].

Research on parent–child interpersonal synchrony suggests that certain conditions originating in either or both the mother and child may impact the development of synchrony [46, 48, 50, 57]. These conditions can include infant prematurity, maternal depression, or autism. In general, with interpersonal synchrony being present but reduced in autistic individuals in comparison to non-autistic individuals. There is evidence that the capacity to synchronise the timing of behaviour with that of others is compromised in autistic individuals and this has been demonstrated across a number of domains including motor, conversational, physiological, and neural synchrony [57, 58]. For example, when carrying out motor tasks such as coordinating the swinging of handheld pendulums or coordinating rocking in a chair with their parents, autistic children have reduced capacity for entrainment in comparison to non-autistic children [58, 59]. From a family system's perspective [60], particularly in parent–child relationships, it is likely that an individual with an atypical circadian rhythm would disrupt the circadian rhythms of cohabiting family members. For example, parents of autistic children often report their sleep is disrupted as a result of their caregiving responsibilities [61]. This is an important area of research as evidence supports that when social cues and circadian clocks become misaligned within the family there can be negative consequences at an individual [62, 63] and family level [64, 65].

This compromised capacity to entrain to social signals at high frequencies (e.g., with rhythms of seconds or minutes) is a consistent finding in autistic individuals and could indicate a general disruption of timing in this condition [57, 58]. This would be consistent with the compromised circadian timing that is also a common trait in autistic individuals [9–12]. The contribution of social entrainment to the disruption of circadian rhythms is rarely considered and it is not known whether the compromised synchronisation of behavioural rhythms of autistic individuals extend to rhythms with circadian or 24-h periods. The parallels between the reduced capacity of autistic individuals to synchronise their behaviour with the timing of social cues, and their disrupted circadian timing have not been explored.

As autism and circadian rhythm regulation are both highly heritable traits, genetic studies have identified shared pathways and candidate genes that implicate disrupted biological timing in the neurodevelopmental architecture of autism [66–68]. Family-based research provides a critical lens through which to examine these shared genetic contributions, particularly by assessing circadian synchrony, sometimes regarded as the alignment of behavioural and physiological rhythms among cohabiting individuals. Synchrony may represent a significant phenotype that reflects underlying circadian mechanisms and social entrainment processes, both of which are altered in autism [58, 59]. Characterizing how synchrony is expressed and measured within families, especially those including autistic individuals, can help clarify whether disrupted circadian timing is a consequence of autism or a contributing factor to its development. Moreover, understanding synchrony in a family context may reveal early indicators of neurodevelopmental risk and inform interventions aimed at improving sleep, behavioural regulation and overall family well-being.

Given the lack of research on this topic, the aims of this scoping review are to (1) determine the extent, nature, and range of the literature available on the synchronisation of circadian rhythms in families with and without autistic children, (2) to map the existing literature in this field and (3) to identify any gaps in the literature. At the time of writing this scoping review, there were no known previous reviews conducted on this topic.

## Main text

A scoping review was conducted using the framework devised by Arksey and O’Malley [69] and refined by Levac et al. [70]. The scoping review was reported using the Preferred Reporting Items for Systematic reviews and Meta-Analyses extension for Scoping Reviews (PRISMA-ScR) checklist.

### Protocol

The research questions, inclusion and exclusion criteria, and process for conducting the review (stages/division of work) were predefined in a protocol, which was developed and agreed upon by two researchers (CW and AB).

### Eligibility criteria

There were two groups included in this scoping review; (1) families, dyads, triads, and cohabitants comprised only of non-autistic individuals, and (2) families, dyads, triads, and cohabitants comprised of one or more autistic individuals. Studies were excluded if autistic individuals did not have a confirmed clinical diagnosis e.g., diagnosis made by a clinical psychiatrist/psychologist using appropriate assessments and tools (e.g., Autism Diagnostic Observation Schedule (ADOS) or Autism Diagnostic Interview (ADI)).

Only peer-reviewed published literature was included to optimise the quality of the data in this review. This included cohort, case control, longitudinal, and cross-sectional studies. Publications were only included if they were available in the English language. There was no exclusion based on the year of publication to ensure all relevant literature was captured. Non-peer reviewed literature, unpublished literature, books, theses, reviews, and grey literature were excluded.

Studies with the following outcomes were included; (1) studies examining circadian rhythm synchrony (assessment of behavioural and/or physiological measures over 24 h/multiple days so that 24-h periods within the dataset could be detected) within cohabiting individuals, mother–child and father-child dyads, mother, father and child triads, siblings or families comprised of only non-autistic individuals or one or more autistic individuals. Studies were excluded if the synchrony examined was not circadian (24-h), for example rhythms with periods of seconds or minutes.

### Information sources and literature search

An initial computerised search of four databases (Scopus, PubMed, CINAHL and PsycInfo) was conducted in November 2021 using relevant search terms and search strategies which were refined and agreed upon by two researchers (CW and AB) prior to conducting the searches. Two separate searches were conducted in each database, one search to identify studies examining circadian rhythm synchrony within families comprised of only non-autistic individuals and another to identify studies examining circadian rhythm synchrony within families with one or more autistic individuals (see supplementary section). These searches were conducted by one researcher (AB). The reference lists of relevant literature were hand searched by two researchers (CW and AB) to identify any additional publications for inclusion. Handsearching was conducted continuously until January 2024 in order to identify any new relevant publications.

### Selection of sources of evidence

**Fig. 1:**
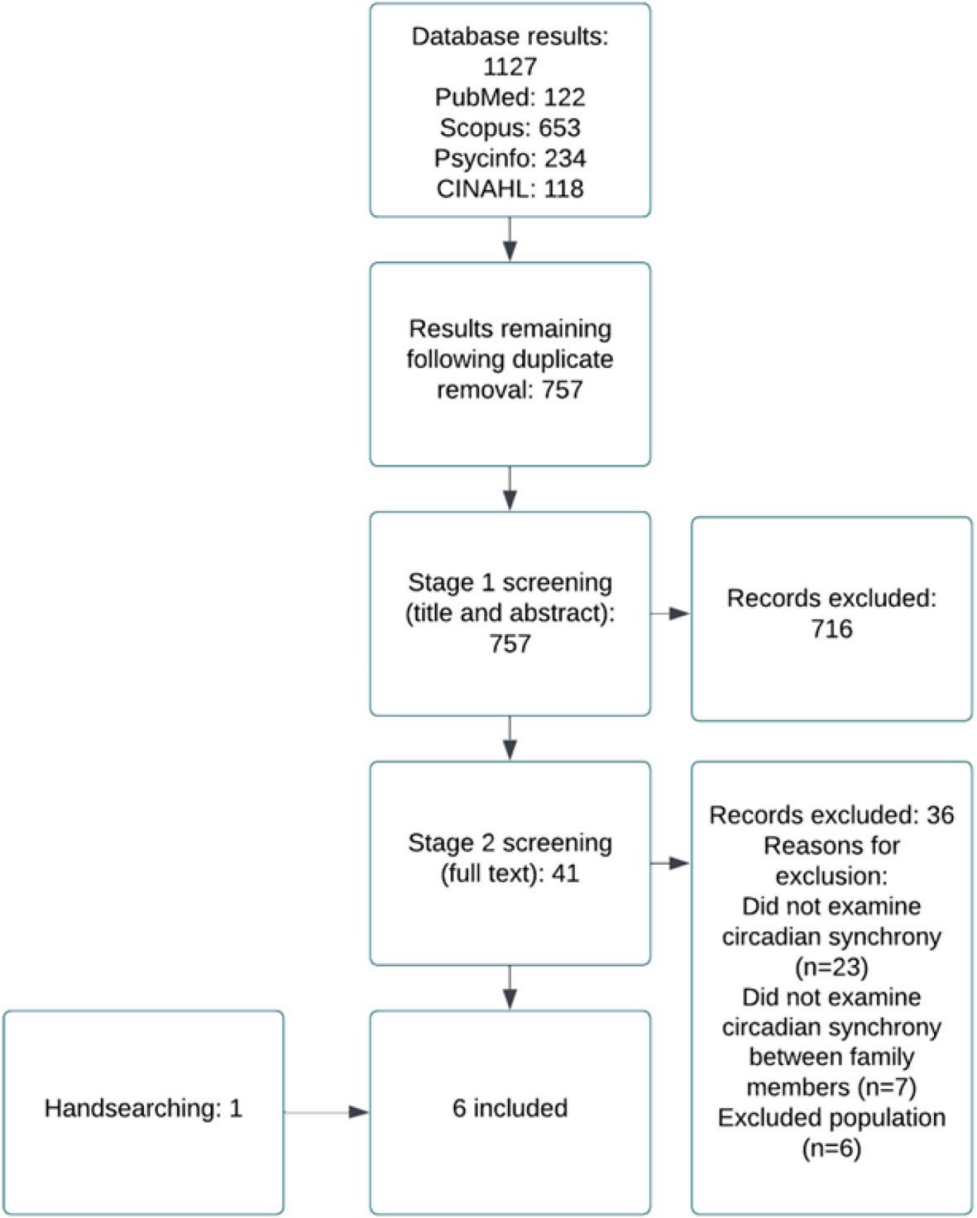
Flow chart of study selection and inclusion for studies examining the synchronisation of circadian rhythms in families with autistic children

**Fig. 2:**
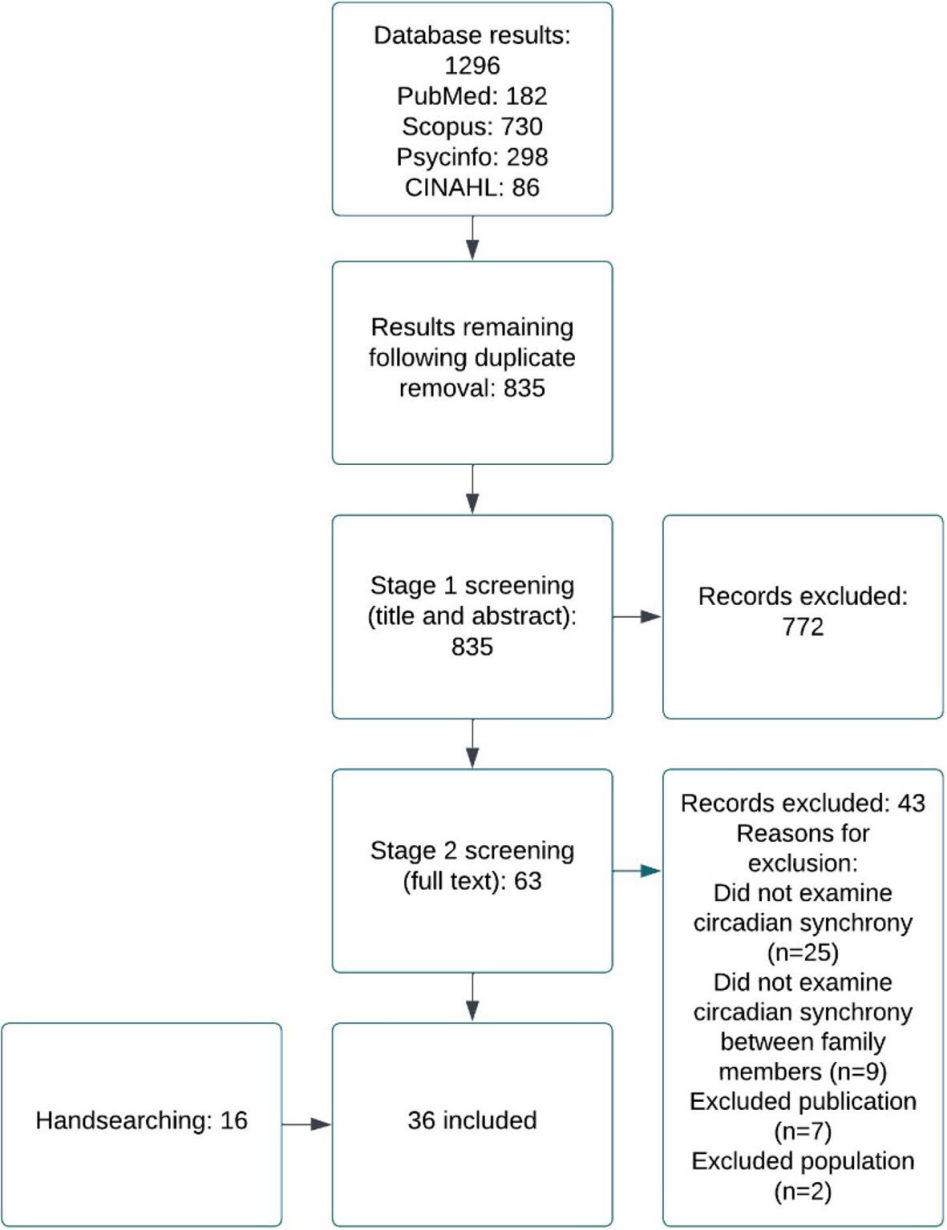
fFlow chart of study selection and inclusion for studies examining the synchronisation of circadian rhythms in families with non-autistic children

The literature identified by the database searches was screened in two phases (please see Figs. 1 and 2). Phase one involved screening based on title and abstract and phase two involved screening the full text articles. Screening was conducted independently by two researchers (CW and AB). The two researchers were blind to each other's decisions until screening was complete, at which point any disagreements were resolved by discussion and involvement of a third reviewer (LML) if necessary.

### Data charting process and data items

Data were charted independently by two researchers (CW and AB) using predefined data extraction tables. Charting data was an iterative process, and the data extraction template was continuously updated throughout the process. Data items of interest included citation information, the objective of the study, the population, the age range of participants, between whom circadian synchrony was measured (e.g., mother-infant synchrony), measures of synchrony, study design, statistics used to parameterise synchronisation, comparison/control, and outcomes/results of the study. For studies that included autistic individuals, data was gathered on how autism diagnoses were confirmed.

### Critical appraisal of individual sources of evidence

The included studies did not undergo critical appraisal which is in line with the scoping review framework that was followed [70].

### Synthesis of results

Results were organised into a narrative synthesis including a descriptive numerical summary and thematic analysis.

### Community involvement statement

This research was reviewed by a neurodiverse team member for greater clarity, understanding, and language inclusivity as informed by the autistic community. Based on this feedback, we revised terminology throughout the manuscript to avoid deficit-based language, for instance, replacing “impairments” with “differences” and “symptoms” with “traits.” We also adjusted the framing of comparison groups, using “non-autistic” instead of “neurotypical” or “control,” and clarified descriptions of behavioural characteristics to better reflect autistic perspectives. These changes were made to ensure respectful representation and improve accessibility for a broader audience, including autistic readers and families.

## Results

### Selection of sources of evidence

The searches yielded a total of 2,423 results. The screening, inclusion and exclusion processes of these results are available in Figs. 1 and 2. An additional 17 studies were identified via handsearching.

### Characteristics of individual sources of evidence

A total of 42 studies, published between 1994 and 2023, were included in this review. Thirty-six studies examined the synchronisation of 24-h patterns in physiology and/or behaviour in families with non-autistic children [71–106] and six studies examined the synchronisation of 24-h patterns in physiology and/or behaviour in families with autistic children [107–112].

Among the six studies that focused on the synchronisation of 24-h patterns in physiology and/or behaviour in families with autistic children, sleep was the only measure investigated. For detailed information on the characteristics of these studies please see Table 1 and Supplementary Table 1, respectively. One study compared the sleep timing and sleep problems of autistic children, their non-autistic siblings, and non-autistic children [107], two studies focused on the relationship between mother and child sleep timing [108, 109] and three studies focused on the relationship between parent (mother and/or father) and child sleep timing [110–112]. All studies used self-reported measures to assess synchrony such as questionnaires, visual analogue scales, checklists, or sleep–wake diaries [107–112]. Three studies also used actigraphy for either seven or 14 nights consecutively [108, 110, 112].

**Table 1:** A comparison of the characteristics of studies examining circadian synchrony in families with autistic children and in families with non-autistic children

	Studies including families with autistic children	Studies including families with non-autistic children
Publication range	2008–2021	1994–2023
Study types	3 Case–control studies 1 Cross-sectional correlation study 2 Pilot studies	16 cross-sectional studies 13 longitudinal studies 4 pilot studies 1 observational 1 cohort 1 case–control
Age range	3–18 years old	2 weeks old to 16 years old
Participant numbers (range)	17 dyads-180 dyads	3 mother-infant dyads-1077 parent–child dyads
Measures of synchrony	All studies used self-reported measures to assess synchrony (e.g., questionnaires, visual analogue scales, checklists, or sleep–wake diaries) 3 studies also used actigraphy for either 7 or 14 nights consecutively	23 studies used actigraphy or accelerometery with or without sleep diaries, logs, and questionnaires 7 studies used only subjective measures recorded by participants (e.g., diaries and self-reported questionnaires and scales) 4 studies used cortisol samples 1 study measured 6-sulfatoxymelatonin in urine 1 study used polysomnography
Synchrony within the family	3 mother/father-child synchrony 2 mother–child synchrony 1 sibling synchrony	18 mother–child/infant synchrony 14 parent–child/infant synchrony or mother, father and child synchrony 1 mother-infant and mother-partner synchrony 1 mother-infant and twin-twin synchrony 1 parent–child and twin-twin synchrony 1 twin-twin synchrony
Type of synchrony	6 studies examined the synchrony of sleep within families	31 studies examined sleep/rest/activity/chronotype synchrony within families 4 studies examined cortisol synchrony within families 1 study examined 6-sulfatoxymelatonin synchony in families

Across the 36 studies that focused on the synchronisation of 24-h patterns in physiology and/or behaviour in families with non-autistic children, biomarker secretion, activity and sleep timing were the only measures investigated. For detailed information on the characteristics of these studies, please see Table 1 and Supplementary Table 2. Four studies examined the timing of cortisol secretion in families with non-autistic children [71, 88, 95, 103]. All studies examined parent (mother and/or father) and child cortisol synchronisation across multiple timepoints. One study compared diurnal salivary cortisol output and maternal-infant cortisol timing synchrony in low and high socio-economic status (SES) mother-infant dyads [71], one study examined the concordance between mother-infant diurnal cortisol secretion in mothers with major depressive disorder (MDD) and mothers without depression [88], one study compared the timing of diurnal cortisol secretion between mother–child pairs in mothers with and without anxiety [103], and one study examined correlations of daily cortisol secretion patterns between mothers, fathers and their children [95]. One study compared the circadian rhythm of 6-sulfatoxymelatonin in urine for two groups of infants (breast and formula fed infants) and their mothers and measured the circadian rhythm of tryptophan in breastmilk [72].

Twelve studies examined the synchronisation of 24-h rest-activity or physical activity patterns in families with non-autistic children [73, 76, 77, 85, 91, 92, 96–98, 101, 104, 105]. Eight studies assessed the synchronisation of 24-h patterns in mother–child rest-activity or activity [73, 76, 77, 85, 91, 97, 98, 101] and four studies assessed the synchronisation of 24-h patterns in parent–child (mother and/or father) rest-activity or activity timing [92, 96, 104, 105]. All studies used either actigraphy/accelerometery [73, 76, 77, 85, 91, 92, 96–98, 101, 104, 105] with or without sleep diaries or logs to investigate activity synchrony.

Nineteen studies assessed the synchronisation of 24-h patterns in sleep in families with non-autistic children [74, 75, 78–84, 86, 87, 89, 90, 93, 94, 99, 100, 102, 106]. Nine studies assessed parent–child sleep timing synchrony (mother and/or father) [74, 75, 79–81, 84, 90, 93, 102], six studies assessed mother–child sleep timing synchrony across 24 h [82, 83, 86, 87, 99, 100], one study assessed mother–child and mother-partner sleep timing synchrony [106], one study assessed mother-infant and twin-twin sleep timing synchrony [78], one study assessed parent (mother and/or father)-child and twin-twin sleep concordance [89] and one study assessed twin-twin sleep concordance [94]. Eleven studies used actigraphy/accelerometery with or without sleep diaries, logs, and questionnaires [78, 79, 81, 86, 87, 89, 90, 93, 94, 100, 102], seven studies used subjective measures of sleep e.g., questionnaires and sleep–wake diaries or sleep logs [74, 75, 80, 83, 84, 99, 106] and one study used polysomnographic recordings in a lab-based setting [82].

### Results of individual sources of evidence

Detailed information on the results of each study in relation to our review questions and objectives is available in Supplementary Table 3 and Supplementary Table 4.

### Synthesis of results

#### Circadian synchrony in families with autistic children

One study demonstrated that autistic children and their non-autistic siblings experienced more sleep problems and maintained more rigid sleep schedules than non-autistic children [107]. Other studies showed associations between mother and child sleep (e.g., longer maternal sleep duration was linked to longer child sleep duration in ‘good sleeper’ dyads) [108], and child’s sleep being a predictor of maternal sleep quality and stress [109]. The relationship between child’s sleep and maternal mental health remained after controlling for the behavioural traits related to autism [109]. Two studies identified that parents of autistic children reported more sleep problems in comparison to parents of non-autistic children. Childrens’ sleep problems were associated with parents’ sleep problems (e.g., parents who reported more sleep problems in their children reported poorer sleep quality for themselves) and child sleep variables were associated with parental depressive symptoms [111, 112]. However, another study that examined parent–child sleep identified no correlation between parent and child sleep variables [110]. Table 2 presents an overview of the results of these studies in relation to our review aims. It is important to note that none of the included studies involving autistic participants stratified findings by cognitive functioning, autism severity, or co-occurring intellectual or developmental disabilities and co-occurring mental health disorders. As such, the synthesis presented here reflects aggregated data across heterogeneous autism profiles, and interpretations should be considered in light of this limitation.

**Table 2:** Results of studies that examined sleep synchrony in families with autistic children

Research study title	Measure(s) of synchrony	Results: Synchrony of circadian rhythms	Evidence: Synchrony of circadian rhythms within the family
Familial sleep and autism spectrum disorder: a pilot actigraphy study of sleep quality, quality of life and psychological distress Leader et al 2021 [110]	Actigraphy	There was no relationship between child actigraphy variables and parent actigraphy variables (bedtime, get up time, time in bed, total sleep time, sleep onset latency, sleep efficiency, wake after sleep onset, number of wake intervals)	Mother–child: No Father-child: No Siblings: NA Mother-father: NA
Concordance of Mother/Child Sleep Patterns Using Actigraphy: Preliminary Findings Goldman et al 2014 [108]	Actigraphy	Autistic children were classified into ‘good sleepers’ and ‘poor sleepers’ based on parental reports Positive associations were found between ‘good sleepers’ mothers and their children for bedtime and total sleep time ‘Poor-sleepers’ mothers and their children showed associations for sleep efficiency, fragmentation, wake after sleep onset and percentage wake bouts Mothers of non-autistic children and their children showed associations for total sleep time When all groups were combined there were significant associations between mothers and their children in terms of bedtime, total sleep time and sleep fragmentation	Mother–child: Yes Father-child: NA Siblings: NA Mother-father: NA
Relationship between children's sleep and mental health in mothers of children with and without ASD Hodge et al 2013 [109]	Questionnaires	Children’s sleep significantly predicted maternal mental health, maternal sleep quality, and maternal stress There was a stronger relationship between children’s sleep and mother’s own sleep and maternal stress for mothers of non-autistic children	Mother–child: Yes Father-child: NA Siblings: NA Mother-father: NA
Sleep problems among Taiwanese children with ASD, their siblings and typically developing children Chou et al 2012 [107]	Questionnaires	Autistic children and their non-autistic siblings had more fixed sleep schedules and more sleep problems compared to the non-autistic comparison group Autistic children and non-autistic siblings of autistic children tended to wake up later on weekdays than non-autistic comparisons. Autistic children rose earlier at the weekends than non-autistic siblings and non-autistic comparisons There was no significant difference in bedtime or total sleep time between the three groups	Mother–child: NA Father-child: NA Siblings: Yes Mother-father: NA
Factors associated with depressive symptoms in parents of children with autism spectrum disorders Meltzer 2011 [112]	Actigraphy, sleep diary, questionnaires	Children with shorter sleep time by actigraphy had more maternal reported sleep disruptions No association was found for child objective sleep quality and paternal reports of child sleep disruptions Significant associations were found between child sleep disturbances and parent sleep quality	Mother–child: Yes Father-child: Yes Siblings: NA Mother-father: NA
Sleep problems of parents of typically developing children and parents of children with ASD Lopez-Wagner et al 2008 [111]		Parents of autistic children reported that they experienced more sleep problems than parents of non-autistic children Significant correlations were found between parents’ reports of their children’s sleep problems and their own sleep problems for both autism and community groups	Mother–child: Yes Father-child: Yes Siblings: NA Mother-father: NA

*Yes* Synchrony noted, *No* Synchrony not noted, *NA* Not assessed/examined in this study

#### Circadian synchrony in families with non-autistic children

The synchronisation of patterns in biomarker timing within the family was evident in all studies (Table 3).

**Table 3:** Results of studies that examined biomarker synchrony in families with non-autistic children

Research study title	Measure(s) of synchrony	Results: Synchrony of circadian rhythms	Evidence: Synchrony of circadian rhythms within families
Mother–child adrenocortical synchrony; Moderation by dyadic relational behaviour Pratt et al 2017 [88]	Diurnal cortisol was collected over two consecutive weekend days. Three samples were taken on each day from mother and child	Maternal cortisol across the three measurements was significant in predicting child cortisol beyond the effect of diurnal variation Maternal depression was not found to moderate cortisol linkage The increased dyadic reciprocity observed in mother–child interaction was associated with reduced coupling of mother and child diurnal cortisol release Increased child daily cortisol secretion was associated with stronger coupling of mother and child diurnal cortisol release	Mother–child: Yes Father-child: NA
The effects of SES on infant and maternal diurnal salivary cortisol output Clearfield et al 2014 [71]	Samples were collected over a single day. Saliva samples were taken three times throughout the day – morning, afternoon and evening	Low SES infants had overall higher cortisol levels than high SES infants High SES dyads were marginally correlated in the morning, significantly correlated in the evening, and not correlated in the afternoon Low SES dyads were not significantly correlated at any time of the day and all correlations were negative. They were increasingly negatively correlated in the afternoon and evening, suggesting that the build-up of daily stress is related to more divergence Overall, more synchrony in high SES dyads than low SES dyads	Mother–child: Yes Father-child: NA
Exploring patterns in cortisol synchrony among anxious and nonanxious mother and child dyads: a preliminary study Williams et al 2013 [103]	At home saliva samples over two consecutive days, 3 times per day (at waking, 30 min after waking, and at bedtime)	There was a significant association between basal cortisol of children and basal cortisol of mothers	Mother–child: Yes Father-child: NA
Comparisons between salivary cortisol levels in six-months-olds and their parents Stenius et al 2008 [95]	Saliva samples were collected in the morning, afternoon and evening	Strong correlations between cortisol levels in mother and infant samples in the morning, afternoon, and evening Weaker correlations in cortisol levels between infant and father’s samples and only in the afternoon and evening samples. Morning father and infant cortisol levels were not associated with one another	Mother–child: Yes Father-child: Yes
The circadian rhythm of tryptophan in breast milk affects the rhythms of 6-sulfatoxymelatonin and sleep in newborn Cubero et al 2005 [72]	Urine samples were taken over a 24-h period to measure 6-sulfatoxymelatonin Breastmilk was collected to assay the amino acid tryptophan Actigraphy for a seven-day period to assess sleep patterns	The circadian rhythm of 6-sulfatoxymelatonin in exclusively breastfed infants was influenced by the rhythm of tryptophan in their mother's milk	Mother–child: Yes Father-child: NA

*Yes* Synchrony noted, *NA* Not assessed/examined in this study, *SES* Socioeconomic status

All studies found positive associations between mother–child activity timing [73, 76, 77, 85, 91, 97, 98, 101], with one study reporting that when mother–child activity rhythms were not in sync there was a significant effect on maternal mental health [91].

Mixed results were found in the studies that examined the synchronisation of 24-h patterns in parent–child (mother and/or father) activity. Parent–child activity levels were correlated in all parent–child dyads, including fathers in one study [96]. Two other studies also found correlations between mother, father, and child activity (e.g., nocturnal activity and onset of daytime activity), but they showed greater correlations between mother–child compared with father-child dyads [104, 105]. Finally, another study showed no evidence of father-infant synchrony, however there was synchrony between mother-infant day and nighttime activity. Any observed differences in mother-infant synchrony were associated with infant age, with greater synchrony in younger infants and reduced alignment in older infants, particularly at night [92]. Table 4 presents an overview of the results of these studies.

**Table 4:** Results of studies that examined activity synchrony in families with non-autistic children

Research study title	Measure(s) of synchrony	Results: Synchrony of circadian rhythms	Evidence: Synchrony of circadian rhythms within families
The influence of feeding method on a mother's circadian rhythm and on the development of her infant's circadian rest-activity rhythm Kikuchi et al 2020 [77]	Actigraphy and diaries	The circadian rest-activity rhythm of the breast-fed infants had started at the 2nd-3rd week and was clearly present in the 6th week. The rest-activity rhythm of the mixed-fed infants was delayed but established by the 12th week Both groups of mothers kept their own circadian rest-activity rhythm from the 2nd-3rd week to the 12th week. The circadian rhythms of breastfeeding mothers and infants were more regular than that of mixed feeding mothers and infants	Mother–child: Yes Father-child: NA Siblings: NA Mother-father: NA
Accelerometery-Derived Physical Activity Correlations Between Parents and Their Fourth-Grade Child Are Specific to Time of Day and Activity Level Strutz et al 2018 [96]	Accelerometry	Weak to moderate correlations were found between parent and child moderate-vigorous physical activity levels Moderate-vigorous physical activity was significantly correlated in all dyads before school, after school, during evening periods and on weekend days Parents’ moderate–vigorous physical activity levels differed based on their children’s activity levels during the evening	Mother–child: Yes Father-child: Yes Siblings: NA Mother-father: NA
Application of Empirical Mode Decomposition to Mother and Infant Physical Activity Synchronisation of Circadian Rhythms is Associated with Maternal Mental Health Shimizu et al 2018 [91]	Actigraphy	There was a significant association between maternal mental health and desynchronisation of mother–infant circadian rhythms of physical activity Diurnal fatigue and depressive mood scores were significantly and positively correlated with decreased mother–child physical activity synchrony	Mother–child: Yes Father-child: NA Siblings: NA Mother-father: NA
Light and maternal influence in the entrainment of activity circadian rhythm in infants 4–12 weeks of age Thomas et al 2016 [97]	Actigraphy and activity log	All maternal and infant circadian measures for light were highly correlated When maternal light was controlled, there was a significant correlation between maternal and infant activity rhythms	Mother–child: Yes Father-child: NA Siblings: NA Mother-father: NA
Mother–infant circadian rhythm: Development of individual patterns and dyadic synchrony Thomas et al 2014 [98]	Actigraphy and sleep diary	Mothers experienced early disruption of their circadian rhythms following birth, with re-establishment of their circadian rhythm over time Infants demonstrated a developmental trajectory of circadian pattern with increasing mesor, magnitude, amplitude, midpoint of lowest 5 (L5), interdaily stability, and intradaily variability Infants increasingly phase advanced relative to their mother over the study duration Evidence of mother-infant synchrony in increasing correspondence of acrophase	Mother–child: Yes Father-child: NA Siblings: NA Mother-father: NA
Relationship Between Infant and Mother Circadian Rest-Activity Rhythm Pre- and Postpartum, in Comparison to an Infant with Free-Running Rhythm Nishihara et al 2012 [85]	Actigraphy and log	Infants circadian rest-activity rhythms were present at 2 weeks and amplitude increased up to 12 weeks. The infants rest-activity rhythm was established by the 12th week Amplitude of mother's autocorrelogram at 24 h decreased after birth, because of increased night-time waking for infant care. The mother’s circadian rhythm became more regular from the 2nd-12th weeks but did not return to pre-partum levels One infant showed a free-running pattern of activity. This infant’s mother had peaks in her circadian rhythm and showed a split rhythm from the 10th-11th weeks (20- and 26-h peaks). These peaks were also seen in the mother’s late pregnancy autocorrelograms At 12 weeks, circadian rest-activity rhythm peaks were observed for the mother and infant and the infants peak appeared to be earlier than the mothers	Mother–child: Yes Father-child: NA Siblings: NA Mother-father: NA
Development of synchrony between activity patterns of mother-infant pair from 4 to 18 months after birth Doi et al 2011 [73]	Actigraphy and sleep diary	Correlations between mother and infant activity patterns increased from 4–18 months, indicating a consolidation of mother-infant synchrony during this period Results also suggest that synchrony strength is uniquely related to age in days of infants	Mother–child: Yes Father-child: NA Siblings: NA Mother-father: NA
Mother-infant activity synchrony as a correlate of the emergence of circadian rhythm Tsai et al 2011 [101]	Actigraphy and sleep diaries	2–10-week-old infants have beginning circadian rhythms and the acrophase of their activity shows an adult-like phase relationship There was a strong mother-infant correlation in activity as well as a strong correlation between their circadian activity patterns. This suggests that infants are behaviourally entrained to the 24-h day by their mothers Within dyad correlation of activity was associated with greater amplitude and the robustness of the infant rhythm	Mother–child: Yes Father-child: NA Siblings: NA Mother-father: NA
Development of the 24-h rest-activity pattern in human infants Jenni et al 2006 [76]	Actigraphy	An ultradian pattern of rest-activity was predominate in newborns Infants gradually developed a 24-h rhythm with a night preference for sleep and a day preference for activity Within the first few weeks after birth, mothers had increased night-time activity and daytime napping	Mother–child: Yes Father-child: NA Siblings: NA Mother-father: NA
Circadian and Ultradian Time Patterns in Human Behavior: Part 2: Social Synchronisation During the Development of the Infant's Diurnal Activity-Rest Pattern Wulff et al 2001 [104]	Actigraphy	Mother-infant pairs had concordant ultradian frequencies at 1, 2 and 4 months Infants circadian pattern emerged during the first few weeks Synchronisation of mother-infant activity was evident from prenatal to postnatal and from the first to the second month Mother infant pairs were more synchronised than father-infant pairs More correlation between mother-infant activity was associated with the rapid development of entrained circadian rhythms in infants	Mother–child: Yes Father-child: Yes Siblings: NA Mother-father: NA
Circadian and Ultradian Time Patterns in Human Behaviour: Part 1: Activity Monitoring of Families from Prepartum to Postpartum Wulff & Siegmund 2000 [105]	Actigraphy and diary	During pre-pregnancy the circadian period length of women tended to correlate to that of their partners. Joint get-up times and bedtimes were common among partners. Pregnant women spent less time resting and were more active than their partners and non-pregnant women Activity at night increased from the prenatal to the postnatal period in mothers and fathers All mothers’ nocturnal activity coincided with that of their infants’ activity, the fathers were affected by indirect disturbances The majority of the mother-infant pairs showed a high correlation of concurrent onset of daytime activity Mothers had lower amplitude circadian rhythms after birth All infants showed a predominant circadian rhythm between day 8 and 19 after birth	Mother–child: Yes Father-child: Yes Siblings: NA Mother-father: Yes
Time patterns in parent‐child interactions in a trobriand village (Papua New Guinea) Siegmund et al 1994 [92]	Activity monitor	Synchronisation was evident between infants and mothers during the day and night, but no father-infant synchronisation was found. There was a difference in the infant and mother’s rhythmicity depending on the infant’s age In older infants, 5–11 months, there was no synchronisation of parents or sibling’s movements in relation to the infant’s nocturnal activities	Mother–child: Yes Father-child: No Siblings: No Mother-father: NA

*Yes* Synchrony noted, *No* Synchrony not noted, *NA* Not assessed/examined in this study

Five studies identified significant associations between mother–child sleep timing [82, 86, 87, 99, 100] and one study reported no correlations between mother-infant sleep variables (bedtime, wake time and total sleep time) [83]. Of the studies that examined the synchronisation of 24-h patterns in parent–child sleep (mother and/or father) there were mixed results in terms of sleep timing synchrony. One study reported that maternal, but not paternal, circadian preference was associated with children’s sleep timing [84]. Similarly, two other studies observed associations between mother–child sleep (including sleep duration, sleep quality, and chronotype) and mother-partner sleep, but no associations between partner-child sleep timing [79, 80]. Conversely, four studies demonstrated positive correlations between all parent–child (including fathers) sleep measures, including measures of sleep duration, sleep midpoint, night wakening, sleep onset and sleep offset [74, 81, 90, 102]. Notably, in one study, these correlations in sleep timing were higher in mother–child dyads, who made up the majority of the cohort [81]. Another study detected low correlations between parent–child sleep timing and significant differences in several sleep measures [75]. The children included in this study were slightly older in comparison to the children included in the other studies that examined parent–child sleep (age range: 10–16 years). Siegmund et al. [93] documented that infants gradually developed a circadian rhythm from ultradian rhythms over time and that their circadian rhythms became similar to that of adults. Parents’ rhythms also had a strong social component [93]. In the study that examined mother–child and mother-partner sleep timing, infant variables were not significantly correlated with the mother’s sleep–wake cycle and the father’s social rhythm was the only significant predictor of the mother’s sleep wake rhythm [106]. The study investigating twin-twin and mother–child sleep synchrony found associations between twin-twin and twin-mother sleep timing, with mothers’ sleep duration increasing as the twin’s sleep patterns became more synchronised [78]. In the study that examined parent–child and twin-twin sleep synchrony there was concordance between both parent–child and twin-twin sleep measures [89]. Finally, in the study that examined twin-twin sleep, there was evidence of concordance between twin-twin sleep variables, with greater concordance evident in monozygotic twins than in dizygotic twins [94]. Table 5 presents an overview of the results of these studies.

**Table 5:** Results of studies that examined sleep synchrony in families with non-autistic children

Research study title	Measure(s) of synchrony	Results: Synchrony of circadian rhythms	Evidence: Synchrony of circadian rhythms within families
Concordance in parent–child and sibling actigraphy-measured sleep: Evidence among early adolescent twins and primary caregivers Sasser et al 2023 [89]	Accelerometry	There was significant daily and average sleep concordance between parent and child sleep duration (total time asleep) and sleep mid-point (midpoint between sleep onset and offset time) Parent and child sleep efficiency (ratio of time spent asleep to total time in bed, with total time in bed consisting of true sleep and waking episodes) was not significantly related to one another at the daily or average level There was significant average but not daily concordance between parent and child sleep latency (number of minutes to sleep onset from first attempting to fall asleep) There was significant concordance between twin’s sleep duration, sleep efficiency, sleep midpoint and sleep latency at a daily and average level Siblings who slept in the same room had greater concordance of sleep measures	Mother–child: Yes Father-child: Yes Siblings: Yes Mother-father: NA
In or out of sync? Concordance between parent and adolescent sleep varies by family context Sasser and Oshri 2023 [90]	Actigraphy	At a daily level, parent and child sleep duration (total time asleep) and sleep midpoint (midpoint between sleep onset and offset time) were significantly associated There were no significant daily associations between parent and child sleep efficiency (ratio of time spent asleep to total time in bed, with total time in bed consisting of true sleep and waking episodes) On average, parent and child sleep midpoint was significantly associated but parent and child sleep efficiency and sleep duration were not significantly associated Family flexibility was associated with higher concordance in sleep duration and midpoint. Adverse parenting was associated with discordance in sleep duration and efficiency	Mother–child: Yes Father-child: Yes Siblings: NA Mother-father: NA
Actigraphy-measured sleep concordance, night-wakings, intraindividual sleep variability in parents and their children—Associations with childhood sleep disturbances Varma et al 2022 [102]	Actigraphy and questionnaires	Sleep concordance between parent–child ranged between 58–89% (mean: 70.6%). The percentage of times the parent was awake during their child’s waking was significantly higher in comparison to the percentage of times the child was awake during their parent’s wakings Parents of children with sleep disturbances displayed significantly poorer sleep quality, higher wakefulness after sleep onset (WASO), and greater variability in sleep duration and bedtime in comparison to parents of children with no sleep difficulties	Mother–child: Yes Father-child: Yes Siblings: NA Mother-father: NA
A longitudinal study of the links between maternal and infant nocturnal wakefulness Tikotzky et al 2021 [100]	Actigraphy, sleep diaries and questionnaires	There were significant associations between mother and infant sleep at all assessment points (in terms of the amount of night wakenings and time spent awake). The strength of this association declined over time These associations were stronger for maternal reports than actigraphy measures There was no significant difference in the correlation between sleep measures and sleeping arrangements (room-sharing vs solitary-sleeping families)	Mother–child: Yes Father-child: NA Siblings: NA Mother-father: NA
The role of parental circadian preference in the onset of sleep difficulties in early childhood Morales-Munoz et al 2019 [84]	Questionnaires	Maternal circadian preference affected the development of the infant’s circadian rhythm Maternal eveningness was associated with slower circadian rhythm development in infants at 3, 8, 18 and 24 months Maternal eveningness was associated with short sleep duration during the daytime at 8 months, short sleep duration during the night at 3 and 8 months, with long sleep-onset latency at 3, 18 and 24 months, with late bedtime at 3, 8, 18 months and with parent-reported sleep difficulties at 8 and 24 months There was no link between paternal circadian preference and children’s sleep at any time point	Mother–child: Yes Father-child: No Siblings: NA Mother-father: NA
Sleep: population epidemiology and concordance in Australian children aged 11–12 years and their parents Matricciani et al 2019 [81]	Accelerometer and log	Parent–child concordance was evident for all measures including sleep duration (the difference between sleep onset and sleep offset), sleep onset (the start of the first three consecutive minutes scored as sleep), sleep offset (the end of the last five consecutive minutes scored as sleep), day to day variability in duration and efficiency (the percent of minutes scored as sleep between onset and offset) Concordance was strongest for sleep onset and offset and in mother–child pairs (notably, mother–child pairs made up the majority of the participants)	Mother–child: Yes Father-child: Yes Siblings: NA Mother-father: NA
The Transition of Sleep Behaviours in Twin Infants and Their Mothers in Early Infancy Kondo and Takada 2018 [78]	Actigraphy and sleep diaries	Twin infants sleep patterns changed from 3–6 weeks to 8–11 weeks – wake duration decreased, and sleep duration increased. The proportion of time both twins were asleep significantly increased Maternal sleep duration during both infants sleeping was significantly correlated with corrected age. Maternal sleep duration increased with the synchronisation of sleep behaviours between twin infants	Mother–child: Yes Father-child: NA Siblings: Yes Mother-father: NA
Within-Family Relations in Objective Sleep Duration, Quality, and Schedule Kourous and El-Sheikh 2017 [79]	Actigraphy and sleep diaries	Children’s sleep duration and sleep quality were related to their mother’s sleep on the same night and to wake times the next morning Mother’s sleep was influenced by child and partners sleep on the same night Father’s sleep was predicted by their partners sleep but not by their children’s sleep	Mother–child: Yes Father-child: No Siblings: NA Mother-father: Yes
Daily Concordance Between Parent and Adolescent Sleep Habits Fuligni et al 2015 [74]	Checklists and questionnaires	There was significant concordance between parent and child daily sleep time. This concordance was equally strong for both parents and children and appeared to be attributed to concordance between both bed and wake times for parents and their children Concordance between parent and child sleep remained even after controlling for other experiences Concordance was strongest among larger families and those with more supportive parent–child relationships	Mother–child: Yes Father-child: Yes Siblings: NA Mother-father: NA
Genetic and Environmental Contributions to Sleep–Wake Behavior in 12-Year Old Twins Sletten et al 2013 [94]	Actigraphy and sleep diary	There were correlations in sleep habits in both monozygotic and dizygotic twins There were greater correlations in sleep habits (sleep onset, sleep efficiency and sleep fragmentation) in monozygotic than in dizygotic twins There was also a trend of greater correlations in total sleep time and start and end of sleep time in monozygotic than in dizygotic twins, but this was not significant Sleep phenotypes between monozygotic twins were similar	Mother–child: NA Father-child: NA Siblings: Yes Mother-father: NA
In sync with the family: children and partners influence the sleep–wake circadian rhythm and social habits of women Leonhard & Randler 2009 [80]	Questionnaires/scales	Children had a significant effect on women’s sleep–wake cycle and chronotype. Women with children, older women and pregnant women were more likely to be earlier chronotypes Women with children had the lowest social jetlag (difference in timing between weekends and weekday) Synchrony between partners increased in pregnancy and returned to pre-birth levels after the birth of the child Synchrony between the mother and child was stronger than synchrony between the mother and partner Chronotypes were correlated between mothers, partners, and their children. Partners chronotypes were highly correlated and there was a correlation between mother–child chronotypes, but no correlation between partner and child	Mother–child: Yes Father-child: No Siblings: NA Mother-father: Yes
Relationship Between Child Sleep Disturbances and Maternal Sleep, Mood, and Parenting Stress: A Pilot Study Meltzer and Mindell 2007 [83]	Questionnaires/scales	There were no significant correlations between parent and child sleep variables (bedtime, wake time and total sleep time), but significant relationships were found between maternal sleep quality and sleep onset latency in children and between maternal sleep quality and child sleep disruptions	Mother–child: Yes Father-child: NA Siblings: NA Mother-father: NA
Family synchronizers: Predictors of sleep–wake rhythm for Japanese first-time mothers Yamazaki 2007 [106]	Questionnaires/scales	Most infant variables were not significantly correlated with the mother’s sleep–wake rhythm The three significant predictors for first time mothers sleep wake rhythm during the early postpartum period were household income, chronotype during pregnancy and the father’s daily social rhythm during the early postpartum period	Mother–child: No Father-child: NA Siblings: NA Mother-father: Yes
Infant sleep and feeding pattern: Effects on maternal sleep Thomas 2005 [99]	Sleep-activity record	Maternal sleep is driven by infant sleep–wake and feeding patterns Increased feeding time decreased infant and maternal sleep time The length of the infants’ longest sleep period was directly related to duration of maternal longest sleep period and indirectly related to number of maternal sleep episodes	Mother–child: Yes Father-child: NA Siblings: NA Mother-father: NA
Similarities and Differences in Sleep–Wake Patterns Among Adults and Their Children Gau and Merikangas 2004 [75]	Questionnaires	Correlations between parents and children were low in terms of morning/eveningness score, frequency of daytime napping, sleep needed to maintain daytime functioning, bedtime, wake time, duration of sleep during weekdays and bedtime and rise times on weekends There is a significant difference in parent–child sleep variables across school grade levels	Mother–child: No Father-child: No Siblings: NA Mother-father: NA
The development of infants’ circadian rest–activity rhythm and mothers’ rhythm Nisihara 2002 [87]	Actigraphy and log	Infant’s sleep–wake rhythm had already begun in the 3rd week There was a 24-h peak for infants in the 3rd week. The amplitude of this peak was the smallest of all the weeks and the regularity of circadian rhythm was the weakest. From the 6th to the 12th week the amplitude of the circadian rhythm gradually increased The amplitude of the 24-h peak of the mother’s circadian rhythm at the 3rd week was the smallest of all weeks and it increased from the 6th to 12th week. This was influenced by the mother’s movements when taking care of her infant at night There was strong synchronisation of mother’s wakefulness and infant’s movement at night after postpartum weeks 1–6	Mother–child: Yes Father-child: NA Siblings: NA Mother-father: NA
Mothers’ wakefulness at night in the post-partum period is related to their infants’ circadian sleep–wake rhythm Nishihara 2000 [86]	Actigraphy	The mothers’ night-time movements significantly decreased from week 3 to week 12. This was related to their infants developing a circadian sleep–wake rhythm 80% of infants showed a prominent circadian component between weeks 8 and 11. All infants showed a 24-h peak at week 12	Mother–child: Yes Father-child: NA Siblings: NA Mother-father: NA
Activity Monitoring of the Inhabitants in Tauwema, a Traditional Melanesian Village: Rest/Activity Behaviour of Trobriand Islanders (Papua New Guinea) Siegmund et al 1998 [93]	Activity monitor	Infants’ circadian rhythms emerge from ultradian patterns in the first few months of life Rhythmicity of adults was related to the light–dark cycle with a strong social component The mean sleep of younger infants was 9–12 h per day and 7–10 h per day for adults On average, wives slept longer than their husbands	Mother–child: NA Father-child: NA Siblings: NA Mother-father: Yes
Sleep and arousal, synchrony and independence, among mothers and infants sleeping apart and together (same bed): an experiment in evolutionary medicine McKenna and Moscow 1994 [82]	Polysomnography	Co-sleeping infants arise more frequently and with greater overlap with mother arousals. This implies sleep arousals are infant/mother induced Infant sleep stages are altered by co-sleeping – decreased sleep stages 3 and 4 and greater simultaneous overlap with mother sleep–wake cycles Co-sleeping mothers and infants spend more time in the same sleep stage or awake condition	Mother–child: Yes Father-child: NA Siblings: NA Mother-father: NA

*Yes* Synchrony noted, *No* Synchrony not noted, *NA* Not assessed/examined in this study

## Discussion

Autism is associated with a reduced capacity to entrain to the timing cues of others [57–59], which may influence synchronisation within family rhythms and could be linked to the circadian disruption often observed in this condition. Synchrony, or concordance of timing within families is an important factor in child development [43–45], and this construct is likely to be driven by a combination of endogenous timing mechanisms and behavioural responses. However, the relative importance of these factors, or the effect of conditions such as autism on family synchrony is unclear. In fact, Several studies, particularly those examining sleep, activity rhythms and biomarker secretion, reported evidence of family entrainment on child circadian rhythms [71–88, 90–113].

Here, we review evidence for the synchronisation of circadian timing in families and whether this is different in families with an autistic child. We summarise evidence for the synchronisation of biomarkers, sleep, and activity in families, but there is no information on the relative importance of the circadian clock in this synchronisation.

The studies included in this review provide evidence that parents of autistic children synchronise to their child’s sleep patterns [108, 112], that the child’s sleep problems correlate with parental reports of their own sleep problems [111], and that these sleep problems are significant predictors of poor maternal mental health and stress [109]. This is consistent with the literature which shows that sleep problems in one family member can impact other family members [114], that parents of autistic children tend to have more sleep problems [114], and that mothers of children with poor sleep tend to report more mood and stress related issues. In families with non-autistic children, synchronisation of daily patterns in biomarker secretion, activity and sleep was evident across the majority of studies with mother–child dyads, whereas results in relation to father-child activity and sleep timing varied between studies.

While these studies take the first step in exploring circadian timing synchrony in families with autistic children and in families with non-autistic children, there is a paucity of studies of circadian rhythms in a family context and several gaps remain in the existing literature. These gaps are discussed further below, alongside directions for future research which will help to advance work in this field. Our broad search strategy reflects the exploratory nature of this emerging topic and the lack of standardized terminology. Although this approach resulted in a low inclusion rate (~ 1.7%), it is consistent with patterns observed in other scoping reviews [115].

### Gaps in the literature

#### Types of measures investigated

All studies included only focused on one measure. The only measure assessed in families with autistic children was sleep and the only measure assessed in families with non-autistic children was either a specific biomarker (e.g., cortisol), sleep, or activity. Other measures that could be used to assess circadian timing synchrony within families include melatonin secretion, core body temperature and clock gene expression [116]. Examining multiple circadian phenotypes (e.g., sleep and melatonin timing) at once could give a better understanding of circadian rhythm synchrony within families across multiple biological systems and could highlight similarities or differences in synchrony across these systems. Furthermore, this approach of combining a behavioural and an endocrine measure could help to separate behavioural and circadian synchronisation.

#### The age range of participants

The infant stage is the focus of the majority of the studies examining circadian rhythm synchrony in families with non-autistic children. This stage is likely one of the key timepoints for the synchronisation of circadian rhythms within the family, specifically, when the infant entrains to the mother’s or caregiver’s circadian rhythms. Infants are born with poorly developed circadian timing, with more ultradian patterns evident in the newborn stage [76]. However, these circadian rhythms continue to develop postnatally with increases in peak activity levels and rhythm regularity and decreases in nighttime activity levels and rhythm fragmentation evident within the first year of life [117]. While light is the predominant *zeitgeber*, the mother or caregiver dictates the infant’s exposure to light and other zeitgebers (e.g., mealtimes), thus mediating entrainment. The development of the infant’s circadian rhythm is essential for coordinating the timing of their activity with the wider family and has far reaching implications for the development, health, and wellbeing of the infant and the entire family unit. However, there is little research available on how the synchrony of circadian rhythms in families with non-autistic children changes as the child ages and progresses through developmental stages. Previous reviews identified that high frequency behavioural and physiological synchrony over short time frames (e.g., heart rate and skin temperature) continues beyond infancy [118] and it is important to understand this in terms of longer term, circadian rhythm synchrony as well. For example, changes in chronotype occur across the lifespan, with younger children typically having an early chronotype and adolescents shifting towards a late chronotype during puberty [37]. Adolescence is also a time when children develop more autonomy and control and it is important to understand the effect that this shift may have on the family, whether synchrony increases or decreases at this stage, and the implications this has for family functioning.

**Fig. 3:**
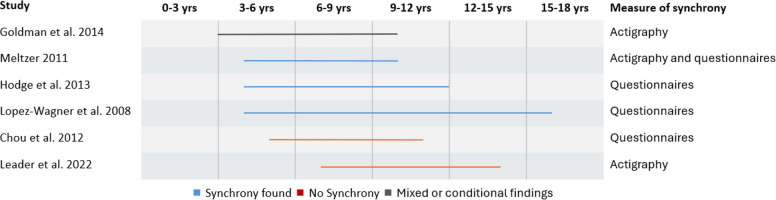
Timeline showing the age range for recruitment of child participants in six studies examining mother–child sleep synchrony in families with autistic children. Color coding indicates whether synchrony was observed (blue), not observed (orange), or mixed/conditional (grey). Mixed/conditional refers to a single study where synchrony was observed only in specific subgroups (e.g., ‘good sleeper’ dyads in [108]). Studies were ordered firstly by lower starting age and secondly shorter range

In contrast, in the studies that included families with autistic children, participants were aged between 3–16 years old, with no research conducted in the infant stage. This is likely because diagnosis typically occurs at a later stage [119] and early diagnosis of autism remains challenging due to a number of factors. However, studies could be conducted in infants with an increased likelihood of developing autism, such as siblings of autistic children [120–123]. Findings regarding mother–child sleep timing synchrony may be influenced by developmental stage rather than solely by autism-related factors. Visual inspection of the age ranges for recruitment and synchrony outcomes across studies suggests this may be a contributing factor (see Fig. 3). Studies reporting synchrony tended to include younger children (e.g., [108, 109]), while those with older samples (e.g., [110]) did not. This pattern aligns with the broader literature, which shows that circadian rhythms and sleep timing shift with age, particularly during adolescence, when chronotype typically moves toward eveningness and sleep autonomy increases [37, 74].

### Influence of co-occurring conditions on sleep in autism

As autism is highly heterogeneous, particularly in terms of cognitive functioning and co-occurring conditions, there are suggestions in the literature that sleep disturbances may be more pronounced in autistic individuals with co-occurring conditions such as ADHD, anxiety or intellectual and developmental disabilities, who show higher rates of insomnia, delayed sleep phase, and night wakings compared to autistic individuals without these comorbidities [124, 125]. The studies included in this review did not stratify findings by cognitive level or report subgroup-specific outcomes. Future research should aim to disaggregate findings by intellectual disability, adaptive functioning and other relevant clinical features to better understand how circadian synchrony may vary across the autism spectrum.

### Synchrony within the family

The studies identified in this review predominantly focused on mother–child synchrony and, in the studies that examined parent–child synchrony (either mothers or fathers), mothers were often the majority [81]. However, our understanding of the synchronisation of circadian rhythms within families is lacking if we do not take into account the influence of the wider family e.g., fathers and siblings. This is particularly important considering that previous research has highlighted that children’s interactions are parent specific [126], which can impact synchrony and affect developmental outcomes in different ways. For example, research has shown that maternal behavioural synchrony is more cyclic (oscillating between low and medium states of arousal, with or without a positive single peak) and socially oriented while paternal interactions are oriented towards the building and organizing of high emotional intensity [126] and that each type of synchrony is important for the development of the child’s social skills. It is possible that this could extend to circadian rhythmicity, with each parent influencing the child’s circadian timing in different ways. Interestingly, there is evidence of greater synchrony between same sex parent–child dyads [126] and this is an area that warrants further research in terms of circadian rhythm synchrony. Collectively, these findings further underscore the relevance of studying circadian timing within family systems, where shared genetic and environmental factors may contribute to synchrony and its disruption in autism.

### Methodological limitations

The studies were limited in their methodology. For example, studies in families with autistic children predominantly used case–control or cross-sectional design and there was no longitudinal data available for families with autistic children. The cross-sectional nature of these studies does not allow for the examination of trajectories of change across developmental stages and it is likely that measures of synchrony change over time and across measures. In particular, the onset of puberty has a marked effect on circadian rhythmicity [37], and it would be interesting to observe the transition of circadian rhythms from childhood to adolescence in autism and in a family context.

The gold-standard method for assessment of the human circadian clock under entrained conditions is the dim light melatonin onset study (DMLO); this test assesses the light-entrained secretion of pineal melatonin, a direct output of the circadian clock. The synchronisation of DMLO within families has not yet been reported to our knowledge and should be prioritised in future research studies of social entrainment. Circadian timing of melatonin in breast milk was estimated by measuring its precursor (tryptophan) in breast milk and corresponding but not phase-aligned circadian patterns in a urinary metabolite of melatonin were reported in their infants [72]. It is not surprising that the phase of maternal breast milk tryptophan and infant urinary 6-sulfatoxymelatonin did not align given the multitude of intermediary metabolic steps that separate relationships between these two analytes. However, these data demonstrate diurnal patterns in melatonin pathways in both mother and infant and support further investigation of their synchronisation and of the role of melatonin as a dietary zeitgeber in this context.

Overall, more longitudinal studies are needed in this area, across developmental periods. However, it is important to note that these can be associated with challenges such as data collection, large participant burden and cost. That said, advances in technology and data analytics have increased opportunities for longer term (over months and years) studies of circadian rhythmicity and future application of these technologies will further the understanding of circadian synchrony within the family unit. In particular, wearable devices have been used with success to assess the circadian timing of sleep and activity in non-autistic children [127] and autistic children [128].

### Limitations of this review

The main limitation of this scoping review is the paucity of research on the synchronisation of circadian rhythms in families with and without autistic children. Additionally, by the lack of stratified data in studies involving autistic participants, which prevents subgroup-level interpretation. The studies included in this review are limited to those identified by our search strategy (search terms, database searching and the handsearching of relevant studies). As such, it is possible that not all relevant studies were identified, particularly because such a variety of terms can be used to describe “synchrony” (e.g., concordance, mutual effect, coregulation, reciprocity). Only studies in the English language were included and grey literature, reviews, commentaries, and books were excluded, which could potentially limit our results. Unlike a systematic review, the included studies did not undergo quality assessment, so studies were not excluded based on methodological rigor. A systematic review was not appropriate in this case it was not in line with the aims of our review.

## Conclusions

Despite growing interest in human chronobiology, the roles of social entrainment and circadian synchronisation within families remain largely unexplored. Nevertheless, it is likely that the circadian clock would entrain to the social *zeitgebers* of another, thus perpetuating rhythms throughout the family. Such entrainment might amplify disrupted circadian rhythms in cohabitating groups and could contribute to the circadian disruption of an entire family unit. In this way, conditions that are associated with disrupted timing, such as autism, could impact the circadian rhythms of the family unit through social entrainment and sleep deprivation. Further research is needed to investigate the relative contribution of behavioural and circadian clock mechanisms to circadian synchronisation in the family context, and how this might differ in families where circadian timing may be atypical such as in families with autistic children. We highlight gaps in the current literature and directions for future research which we hope will spur further research on these topics. Disrupted circadian rhythms should be considered in a family context particularly in studies of autism, a condition that is characterised by both a compromised capacity to entrain to the social cues of others and disrupted circadian timing.

## Supplementary Information

Supplementary Material 1.

## Data Availability

No datasets were generated or analysed during the current study.

## Glossary

**Acrophase**: the peak time of a circadian rhythm, indicating when a biological process reaches its maximum level during a 24-hour period.
**Accelerometery**: the measurement and analysis of acceleration, often done with wearable sensors to monitor and record movements.
**Actigraphy**: a method for monitoring and recording an individual's activity levels and patterns over time using a small, wrist-worn device called an accelerometer.
**Amplitude**: refers to the strength or magnitude of a rhythmic biological process, indicating the difference between its highest and lowest points over a 24-hour period.
**Autocorrelogram**: a graph used in circadian biology to depict regular patterns and rhythms in physiological or behavioural data over time.
**Chronobiology**: the study of rhythmic patterns in biological processes.
**Chronotype**: an individual's natural preference for specific times of the day, reflecting whether they are inclined to be a morning, evening, or intermediate type.
**Circadian**: recurring naturally on a twenty-four-hour cycle, even in the absence of light fluctuations.
**Circadian period**: the duration it takes for a specific biological rhythm or behaviour to complete one cycle within a 24-hour day.
**Concordance**: refers to the similarity or alignment of the timing and characteristics patterns among different people.
**Dim Light Melatonin Onset**: the time in the evening when the pineal gland starts releasing melatonin in response to decreasing light levels. It serves as a marker for the onset of the biological night, aiding in the assessment of circadian rhythms.
**Entrainment**: the synchronization of an organism's internal biological clock with external cues, like the day-night cycle, to regulate physiological functions.
**Free running**: describes the natural, internal rhythm of biological processes when not influenced by external cues like light-dark cycles.
**Interdaily stability**: the consistency and predictability of an individual's daily patterns over consecutive days, reflecting the stability of their circadian rhythm across a 24-hour cycle.
**Intradaily variability**: refers to the fluctuations and irregularities observed within a single day in an individual's activity or physiological patterns, indicating the variability in their circadian rhythm across shorter time intervals.
**L5**: the five hours during a 24-hour duration with least activity.
**M10**: the ten hours during a 24-hour duration with most activity.
**Mesor**: the average level or midpoint of a circadian rhythm over a 24-hour period.
**Phase**: a specific point or stage within the rhythmic cycle of a biological process. It indicates the relative timing of an organism's internal biological events, such as peak activity, hormone secretion, or body temperature, in relation to the external 24-hour day-night cycle.
**Phase advanced**: a shift toward an earlier time in the timing of circadian rhythms or specific events, such as sleep-wake cycles.
**Photoperiod**: refers to the duration of light exposure during a 24-hour period, influencing the regulation of circadian rhythms and biological processes.
**Robustness**: refers to the stability and resilience of the circadian rhythm in the face of internal or external disruptions.
**Social Jetlag**: the misalignment between an individual's natural biological clock and their socially dictated schedule.
**Suprachiasmatic nuclei **: clusters of cells located in the hypothalamus of the brain. These nuclei play a crucial role in regulating the circadian rhythms of an organism.
**Synchronisation**: refers to the alignment or coordination of an organism's internal biological processes with external environmental cues, especially the 24-hour day-night cycle.
**Rhythm fragmentation**: the disruption or irregularity in the timing of biological rhythms, particularly circadian rhythms, leading to inconsistencies in processes like sleep-wake cycles.
**Ultradian**: biological rhythms or processes that occur more frequently than once in a 24-hour period, typically with a frequency higher than the circadian rhythm.
**Zeitgebers**: external cues, like light or social interactions, that synchronize an organism's circadian rhythms with the 24-hour day.
