# Supplementary material for: Synchronisation of circadian timing in families and the impact of autism: a scoping review
Source: J Neurodev Disord. 2026 Feb 25;18:18. doi: 10.1186/s11689-026-09679-z (PMC13040797; doi:10.1186/s11689-026-09679-z)
Supplement: Supplementary file 1 — Supplementary Material 1. [file 11689_2026_9679_MOESM1_ESM.docx]

**SUPPLEMENTARY SECTION**

**Table of contents**

| **Item** | **Page** |
| --- | --- |
| Example of search performed | 2 |
| Supplementary Table 1 | 3 |
| Supplementary Table 2 | 10 |
| Supplementary Table 3 | 37 |
| Supplementary Table 4 | 42 |

**Example search in Scopus for literature relating to circadian synchrony in families with non-autistic children**

( TITLE-ABS-KEY ( clock OR circadian OR chronobiolog* OR melatonin OR sleep* OR rhythm OR pattern* OR bedtime OR diurnal ) AND TITLE-ABS-KEY ( family OR dyad OR triad OR cohabit* OR social OR mother OR father OR parent ) AND TITLE-ABS-KEY ( entrain* OR synchron* OR desynchron* OR zeitgeber ) AND TITLE-ABS-KEY ( child OR infant OR baby OR adolescent ) ) AND ( LIMIT-TO ( LANGUAGE , "English" ) )

**Example search for literature relating to circadian synchrony in families with autistic children**

( TITLE-ABS-KEY ( clock OR circadian OR chronobiolog* OR entrain* OR synchron* OR rhythm* OR melatonin OR desynchron* OR zeitgeber OR sleep* ) AND TITLE-ABS-KEY ( autis* OR asperger’s OR autism ) AND TITLE-ABS-KEY ( dyads OR triads OR cohabit* OR siblings OR stepfamily OR family OR "Parent-infant" OR "Mother-infant" OR "Father-infant" OR "parent-child" OR "mother-child" OR "father-child" ) AND TITLE-ABS-KEY ( child OR infant OR baby OR adolescent ) ) AND ( LIMIT-TO ( LANGUAGE , "English" ) )

**Supplementary Table 1**

*Characteristics of individual sources of evidence including families with autistic children.*

|  | Citation: Title, author(s), year of publication | Objective/purpose of the study | Population (including number of participants) | Mother/Father/sibling Synchrony | Age of autistic participants | Autism diagnosis criteria | Study design | Measures used to assess synchrony (accelerometery, questionnaire etc) |
| --- | --- | --- | --- | --- | --- | --- | --- | --- |
| Sleep Synchrony | | | | | | | | |
|  | Familial sleep and autism spectrum disorder: a pilot actigraphy study of sleep quality, quality of life and psychological distress  Leader et al  2021 (Leader et al. 2022) | To assess objective measures of sleep in autistic children and their parents.  To examine parent and child sleep as factors associated with the stress, anxiety, depressive symptoms, social support, and quality of life of parents of with autistic children. | 21 participants in total - Nine autistic children and their parents (nine mothers and three fathers). | Parent-child sleep synchrony. | 3-18 years old. | Autistic children had an independent diagnosis in accordance with DSM-IV-TR criteria.  Diagnoses were made by an independent psychologist or paediatrician (following a formal diagnostic protocol which employs multiple diagnostic measures).  A caregiver provided the information on the professional diagnosis, the setting/organisation where the diagnosis was made and the professional(s) who made the diagnosis. | Pilot study. | 7-day sleep diary.  Pittsburg sleep quality index (PSQI).  Actigraphy for 7 days and night consecutively. |
|  | Concordance of Mother/Child Sleep Patterns Using Actigraphy: Preliminary Findings  Goldman et al.  2014 (Goldman et al. 2014) | To evaluate the relationship of mother and child sleep with behaviour in autistic children and relate these sleep patterns to the mother’s symptoms of insomnia and daytime sleepiness. | 17 mother-child dyads.  11 autistic children and 6 non-autistic children.  Autistic children were classified into good-sleepers (n=6) and poor-sleepers (n=5).  Autistic children were age and gender matched to non-autistic children. | Mother and child sleep synchrony. | Children ranged in age from 4–10 years. | All autistic children met the criteria for a clinical diagnosis of autism confirmed by the Autism Diagnostic Observation Schedule, as well as a clinical interview incorporating the Diagnostic and Statistical Manual of Mental Disorders, 4th edition.  Children were included if they did not have a history of an intellectual disability, epilepsy or untreated sleep apnoea.  Non-autistic children were excluded if they had an autistic sibling. | Pilot study. | Questionnaires on sleep and daytime sleepiness in mothers: The Epworth Sleepiness Scale and The Insomnia Severity Index.  Sleep-wake diary.  Actigraphy monitor worn for 14 consecutive nights on non-dominant wrist (contained accelerometer).  The Child Behaviour Checklist. |
|  | Relationship between children's sleep and mental health in mothers of children with and without ASD  Hodge et al.  2013 (Hodge et al. 2013) | To explore whether children’s sleep directly impacts mother’s mental health or whether the influence on mental health is delivered via the impact of children’s sleep on the potential mediating variables of maternal sleep and maternal stress. | A total of 180 mother-child dyads.  90 mother-autistic child dyads were selected from a larger dataset of families participating in a separate program of research.  90 age, sex and ethnicity matched controls were recruited from the community. | Mother-child. | Children were 4-12 years old. | Diagnoses of autism were made by qualified mental health professionals – each child was also reviewed or assessed by the referring agency according to regulations of the State Department of Developmental Services. | Case-control. | Gilliam ASD Rating Scale – Second Addition: used to assess symptomology.  Children Sleep Habits Questionnaire: assessed sleep quality.  Parenting Stress Index: evaluates stress in parent-infant systems.  Pittsburgh Sleep Quality Index: used to assess maternal sleep.  The symptom assessment-45 questionnaire: used to measure maternal mental health. |
|  | Sleep problems among Taiwanese children with ASD, their siblings and typically developing children  Chou et al.  2012 (Chou et al. 2012) | To compare the sleep schedules and sleep problems among autistic children, their non-autistic siblings and non-autistic children, and to explore other factors associated with sleep problems. | 110 children who were clinically diagnosed with autism, 125 unaffected siblings, and 110 age-, sex-, and parental education-matched non-autistic children. | Sibling sleep synchrony | The 110 autistic children were aged between 5-13 years old.  The non-autistic siblings were aged 4-13 years old. | The diagnosis of autism was based on DSM-IV criteria for autism and made by board-certified child psychiatrists.  The diagnosis was confirmed by the Chinese Version of the Autism Diagnostic Interview-Revised (ADI-R) in a subsample. | Case-control. | Questionnaires (modified from the sleep habit questionnaire) to survey children’s current sleep problems including dyssomnia (early insomnia, middle insomnia, disturbed circadian rhythm), disordered sleep breathing, parasomnia, and others (including restless leg syndrome, mouth breathing, and frequent daytime inadvertent napping).  Chinese version of the parent bonding instrument.  Pre-natal and peri-natal exposures questionnaire. |
|  | Factors associated with depressive symptoms in parents of children with autism spectrum disorders  Meltzer.  2011 (Meltzer 2011) | The purpose of this study was to examine child and parent sleep as factors associated with depressive symptoms in parents of autistic children and non-autistic children. | Participants included 34 families.  Autistic group: 17 autistic children (77% male), 17 mothers and 11 fathers.  Non-autistic group: 17 non-autistic children (59% male), 17 mothers, 11 fathers. | Parent (mother and father) and child. | Age range: 4-10 years.  Autistic group: mean age of 6.6 years.  Non-autistic group: mean age of 6.4 years. | Autistic children were required to have a documented diagnosis from a physician or psychologist, as well as a score of > 15 (cut-off point for children likely to have autism) on the Social Communication Questionnaire (SCQ).  Children in the non-autistic group were required to have a score of < 15 on the SCQ.  Families from both groups were excluded if a parent/child had a diagnosis of obstructive sleep apnoea, narcolepsy or restless leg syndrome. | Case-control. | An actigraph was worn by all participants (child, mother, father) for 7 consecutive days and nights on their non-dominant wrist. Sleep was determined using the validated Sadeh scoring algorithm.  A daily sleep diary was kept by caregivers during the week that they wore the actigraph.  A daily visual analog scale (VAS) was used by caregivers to record their sleep quality the previous night.  The children’s sleep habit questionnaire was used to determine the child’s sleep behaviour during the previous week.  The Child Behavior Checklist (CBCL) and the Developmental Behaviour Checklist (DBC) were used to determine child behavioural problems.  The Center for Epidemiological Studies–Depression Scale (CES-D) was used to measured depressive symptoms by parents. |
|  | Sleep problems of parents of typically developing children and parents of children with ASD  Lopez-Wagner et al.  2008 (Lopez-Wagner et al. 2008) | To compare parents’ reports of their own sleep problems for both parents of non-autistic children and parents of autistic children and to examine the relation between children’s sleep problems and those of their parents for these groups. | Autism group: 106 parents and their children. 84% of children in the autism sample were male and 16% were female. 48 children had a diagnosis of autism only, 58 children had co-morbidities.  Community group (non-autistic group): 168 parents and children. 55% of children were male and 45% were female. | Parent-child. | Autism group: age range 4-16 years old.  Community group: age range 4-15 years old. | Parents in the autism group each had a child in the program with an  independent diagnosis of autism provided by the referring agency.  The referring agency also provided demographic information and specifics regarding children’s diagnoses. | Cross-sectional correlation. | The Pittsburgh Sleep Quality Index.  Children’s Sleep Habits Questionnaire.  Gilliam ASD Rating Scale, Second Edition. |

**Supplementary Table 2**

*Characteristics of Individual sources of evidence that included only families with non-autistic children.*

|  | Citation: Title, year of publication | Objective/purpose of the study | Population | Type of synchrony | Age of participants | Inclusion criteria/participant characteristics | Study design | Measures used to assess synchrony (accelerometery, questionnaire etc) |
| --- | --- | --- | --- | --- | --- | --- | --- | --- |
| Biomarker synchrony | | | | | | | | |
|  | Mother-child adrenocortical synchrony; Moderation by dyadic relational behavior  Pratt et al.  2017 (Pratt et al. 2017) | The study aimed to examine whether mother-child relational behaviour and maternal psychopathology may moderate the degree of concordance between mother and child's diurnal cortisol. | 97 mothers and their 6-year-old children across two groups; mothers diagnosed with major depressive disorder (n=28) and non-depressed controls (n=69). | Mother-child synchrony. | At assessment at 6 years old, children had a mean age of 6.33 years old, mothers age was 38.88 years and fathers age were 41.04 years. | Recruited only mothers who were healthy, completed high school, were at least 21 years old, above poverty cut-off, were married or cohabitating with the child's father, and whose infants were born at term and were healthy and singleton.  Women with Beck Depression Inventory (BDI) scores in the high end of the depressive symptoms continuum (BDI scores > 11) at birth were recruited for the depressed group, and women in the low end of the depressive symptoms continuum (BDI scores < 8) at birth were recruited for the non-depressed group.  Mothers with mid-level depression scores were excluded between screenings.  Women with high levels of anxiety or eating disorders were also excluded. | Cross-sectional. | Diurnal cortisol collection: Diurnal cortisol was collected over two consecutive weekend days. Three samples were taken on each day from mother and child (a) at awakening, prior to eating, drinking, or brushing teeth, (b) at noon time before eating lunch, and (c) immediately before going to bed prior to teeth brushing.  Beck Depression Inventory – measured at birth, 6 and 9 months.  State-trait anxiety inventory – used at birth, 6 and 9 months.  Maternal psychiatric diagnosis – measured at both 9 months and 6 years. Diagnosis was made using the Structured Clinical Interview for DSM-IV Axis I Disorders.  Mother-child interaction: 10 minutes of mother-child interactions with a set of pre-selected toys were filmed. Interactions were coded with the Coding Interactive Behavior (CIB) manual.  The Dyadic Reciprocity and Dyadic Tension constructs were also used. |
|  | The effects of SES on infant and maternal diurnal salivary cortisol output  Clearfield et al.  2014 (Clearfield et al. 2014) | To compare diurnal salivary cortisol output and maternal-infant synchrony in low and high socio-economic status (SES) mother–infant dyads. | Thirty-two mother–infant dyads.  16 high-SES mothers and infants and 16 low-SES mothers and infants. | Mother-child synchrony.  Synchrony in this case refers to attunement of adrenocortical function between mothers and their infants. | Infants were 6–12 months of age. | Socioeconomic status was based on a needs assessment. | Cross-sectional. | Saliva samples (infant salivary cortisol samples were collected using commercial Salimetrics Infant Swab (SIS) collection kits, and maternal samples were collected using Salimetrics Oral Swab (SOS) collection kits).  Samples were collected over a single day. Saliva samples were taken three times throughout the day – morning, afternoon and evening. |
|  | Exploring patterns in cortisol synchrony among anxious and nonanxious mother and child dyads: a preliminary study  Williams et al.  2013 (Williams et al. 2013) | To test for synchrony between cortisol profiles of mothers and their children, to assess the contribution of anxiety to cortisol concentration at different times during the day, to explore the contribution of anxiety and family environment to the independent diurnal cortisol rhythms of both mother and child, and to explore the dyadic nature of diurnal cortisol levels. | 27 mother-child dyads.  15 children and 17 mothers met the criteria for the primary diagnosis of anxiety. | Mother-infant cortisol synchrony. | Children aged 7-12 years old. | Dyads were excluded from the study if the child or the mother met criteria for a primary diagnosis other than anxiety. | Pilot/exploratory study.  Cross-sectional. | At home saliva samples over two consecutive days, 3 times per day (at waking, 30 min after waking, and at bedtime).  Anxiety Disorders Interview Schedule-IV-Client Version.  Anxiety Disorders Interview Schedule-Parent and Child Versions.  McMaster Family Assessment Device. |
|  | Comparisons between salivary cortisol levels in six-months-olds and their parents.  Stenius et al.  2008 (Stenius et al. 2008) | To examine the correlation between parent (mother and father) and infant cortisol levels. | 51 infants and their parents (mother and father). | Parent and child cortisol synchrony. | Infants were 6 months old. | Not specified.  27 children were recruited from anthroposophic maternity welfare centres.  24 children were recruited from standard maternity care. | Cross-sectional. | Saliva samples were collected in the morning, afternoon and evening and analysed using a radioimmunoassay. |
|  | The circadian rhythm of tryptophan  in breast milk affects the rhythms of  6-sulfatoxymelatonin and sleep in newborn  Cubero et al.  2005 (Cubero et al., 2005) | To compare the circadian rhythm of 6-sulfatoxymelatonin in urine of bottle-fed and breast-fed children, to relate it to the circadian rhythm of tryptophan in breast milk, and to evaluate the effects on the baby’s night-time rest. | 16 healthy infants and their mothers.  8 breastfed infants and 8 formula fed infants. | Mother-child. | Infants aged 12 weeks old. | Healthy infants aged 12 weeks (determined by physical examination and follow up).  Participants took no drugs that would affect 6-sulfatoxymelatonin and tryptophan. levels. | Case-control. | Urine samples were taken over a 24-hour period. This was used to measure 6-sulfatoxymelatonin in urine.  Breastmilk was collected to assay the amino acid tryptophan.  Actigraphy for a seven-day period to assess sleep patterns. |
| Activity synchrony | | | | | | | | |
|  | The influence of feeding method on a mother's circadian rhythm and on the development of her infant's circadian rest-activity rhythm  Kikuchi et al.  2020 (Kikuchi et al. 2020) | To examine the influence of feeding method on the mother's rhythm and on the development of her infant's circadian rest-activity rhythm. | 24 healthy primiparas and their full-term infants.  17 breastfeeding, 7 mixed feeding. | Mother-infant synchrony. | Maternal age (mean) 29.8 years.  Infants were assessed at 2-3 weeks, 6 weeks and 12 weeks. | Infants were healthy, singleton, and vaginally born with uncomplicated deliveries. | Longitudinal. | Actigraphic recordings for infants and mothers using Actiwatches.  Recordings were taken over 3 to 5 consecutive days during the 2nd-3rd week, the 6^th^ week, and the 12th week.  Dairies were also kept - containing mother and infant sleep logs, feeding times, and the times of putting on and taking off the Actiwatches. |
|  | Accelerometery-Derived Physical Activity Correlations Between Parents and Their Fourth-Grade Child Are Specific to Time of Day and Activity Level.  Strutz et al.  2018 (Strutz et al. 2018) | The purpose of this study was to employ high-frequency accelerometery to explore parent–child physical activity (PA) relationships across a free-living sample. | 168 dyads – children and their parents (n=336). | Parent and child synchrony – physical activity. | Mean age of children 9.1 years old.  Mean age of parent 38.8 years old. | All dyads were participating in the Fuel for Fun obesity prevention effort. | Cross-sectional. | 7 days of wrist-mounted accelerometery data. |
|  | Application of Empirical Mode Decomposition to Mother and Infant Physical Activity: Synchronisation of Circadian Rhythms is Associated with Maternal Mental Health  Shimizu et al.  2018 (Shimizu et al. 2018) | To investigate if the synchronisation properties of mother–infant circadian rhythms are associated with maternal mental health during child-rearing periods. | 20 mother-infant pairs. | Mother-infant synchrony. | Infants had a mean age of 7 months.  The mean age of mothers was 33.4 years. | Healthy mother infant pairs. | Cross-sectional. | Actigraphy was used for continuous and simultaneous recordings of mother and infant physical activity over 1 week.  Ecological momentary assessment (EMA) to examine maternal symptoms in daily life. |
|  | Light and maternal influence in the entrainment of activity circadian rhythm in infants 4–12 weeks of age  Thomas et al.  2016 (Thomas et al. 2016) | To investigate the influence of light and maternal activity on early infant activity rhythm. | 43 mother-infant pairs. | Mother-infant activity rhythm synchrony. | Infants were assessed at 4, 8 and 12 weeks. | Healthy, biologic mother-infant pairs, absent of complications prior to or following birth in either mother or infant. | Longitudinal cross-sectional study. | Infant and maternal activity and photopic light were monitored continuously at 1 min intervals over 3 consecutive days using actigraphy.  Activity was verified with a log of both maternal and infant activity completed by the mother. Mothers also recorded periods of external motion (such as holding).  Aggregate maternal and infant minute-by-minute light were graphed against solar light pattern obtained from radiometric recordings by the campus Atmospheric Science Department. |
|  | Mother–infant circadian rhythm: Development of individual patterns and dyadic synchrony  Thomas et al.  2014 (Thomas et al. 2014) | To examine the longitudinal pattern of maternal–infant circadian rhythm and rhythm synchrony. | 43 mother-infant pairs. | Mother-infant. | 4, 8 and 12 weeks. | Healthy mother-infant pairs.  Inclusion criteria: gestation 38–42 weeks, singleton birth, maternal age 18 to 40 years.  Maternal exclusion factors included: depression at time of enrolment, history of sleep disorder, medications altering circadian rhythm, chronic health problems, ante- and postpartum complications.  Infants experiencing pre- or postnatal complications were excluded. | Longitudinal. | Actigraphy: mothers and infants were recorded continuously over a 72-hour period.  Sleep diary: mothers recorded their own and their infants sleep-wake pattern in 15-minute epochs.  Periods where the infants was held or exposed to external motion were also recorded, alongside feeding times. |
|  | Relationship Between Infant and Mother Circadian Rest-Activity Rhythm Pre- and Postpartum, in Comparison to an Infant With Free-Running Rhythm  Nishihara et al.  2012 (Nishihara et al. 2012) | To learn whether a mother’s 24-h rhythm in late pregnancy influenced the development of her infant’s circadian rest-activity rhythm. | 10 pregnant mothers and infants. | Mother-infant. | Late pregnancy and 2-, 6-, and 12-weeks post-partum. | Healthy first-time mothers and their infants.  The family consisted of only infants and their parents.  All infants were vaginally born at full-term. | Longitudinal | Actigraph recordings were made with a sampling rate of 30s over 3-5 days during the 2^nd^, 6^th^ and 12^th^ weeks. One mother recorded continuously from the 10^th^-12^th^ week.  Mothers recorded their activity in late pregnancy – 33^rd^ and 36^th^ weeks of gestation.  Mothers kept a daily log of her, and her infants sleep and feeding times. |
|  | Development of synchrony between activity patterns of mother-infant pair from 4 to 18 months after birth  Doi et al.  2011 (Doi et al. 2011) | To measure motor activities of mother–infant pairs for 3.5 consecutive days by actigraphy and to investigate the developmental course of mother–infant synchrony from 4-18 months after birth. | 48 mother-infant dyads. | Mother-infant synchrony.  Synchrony of activity patterns from 4-18 months after birth. | Infants were aged between 4 and 18 months.  Mean age of mothers was 32.7 years. | Healthy full term vaginally delivered infants and their mothers.  None of the participants had been diagnosed as having neurological disorders, and no abnormal delivery was reported.  All mothers were full-time housewives at the time of participation. | Cross-sectional. | Actigraphs, sleep diaries (recorded start and stop of actigraph recording, time infant slept/woke/fed/was bathed) and questionnaires (included questions on frequency of being woken at night).  Measurements were taken over approximately 3.5 days. |
|  | Mother-infant activity synchrony as a correlate of the emergence of circadian rhythm  Tsai et al*.*  2011 (Tsai et al. 2011) | To examine the activity level and circadian pattern in mothers and infants. | 22 healthy mothers and their infants. | Mother-infant synchrony. | Infants were aged between 2-10 weeks old. | Eligible mothers were >18 years of age, biological first-time mothers, primary caregivers of their babies, not experiencing postpartum depression, and had no medical complications during pregnancy and the postpartum period.  Eligible infants had been born a singleton in a vaginal birth at between 38- and 42-weeks’ gestation, were between 2- and 10-weeks’ postnatal age and were the first-born child in a family with only one child. | Cross-sectional study. | Actigraphy monitors (In-home) for 7 days. 30s intervals.  Sleep diary (sleep/activity record) for mothers to record her infant’s and her own sleep–wake times and the times when the monitor was removed and when the infant was exposed to external motion. These were recorded in 15-minute intervals.  Mothers also reported their sleeping arrangements with their infant every night.  Edinburgh Postnatal Depression Scale. |
|  | Development of the 24-h rest-activity pattern in human infants  Jenni et al.  2006 (Jenni et al. 2006) | To investigate the development of the 24-h rest-activity pattern in human infants under naturalistic conditions as assessed by continuous actigraphy and to estimate the emergence, the full expression and the rate of development of the 24-h component using periodogram analysis. | 7 infant and mother pairs were recorded for:  4 months (n=1),  6 months (n = 5)  12 months (n = 1) | Mother-infant synchrony. | From birth to 4 (n=1), 6 (n=5) and 12 months (n=1) of age. | All infants were delivered without peri- or post-natal complications.  At birth, infants were physiologically, and neurologically normal.  Infant-parent bedsharing did not occur.  The infants remained healthy during the study period. | Longitudinal. | Rest-activity patterns were recorded continuously using actigraphy. |
|  | Circadian and Ultradian Time Patterns in Human Behavior: Part 2: Social Synchronisation During the Development of the Infant's Diurnal Activity-Rest Pattern  Wulff et al.  2001 (Wulff et al. 2001) | To investigate how activity patterns of the entire family entrain to one another and how the infant entrains to the environment. | 12 families – father, mother and child. | Mother, father and infant synchrony. | 37th week of gestation to 4 months. | First born child. | Longitudinal study. | Families were continuously recorded in three series of three weeks from 37 weeks gestation to four months after birth (1^st^ to 3^rd^ week, 7^th^-9^th^ week, and 13^th^-15^th^ week) using Actiwatch units. |
|  | Circadian and Ultradian Time Patterns in Human Behaviour: Part 1: Activity Monitoring of Families from Prepartum to Postpartum    Wulff & Siegmund  2000 (Wulff and Siegmund 2000) | To examine activity-rest patterns during late pregnancy and the first four months after birth in mothers, infants and fathers. | 7 families – mother, father and infant. | Mother, father and infant synchrony. | 37th week of gestation to 4 months. | First born child. | Longitudinal study. | Families were continuously recorded in three series of three weeks from 37 weeks gestation to four months after birth (1^st^ to 3^rd^ week, 7^th^-9^th^ week, and 13^th^-15^th^ week) using Actiwatch units.  A diary was kept to record household routines, parental activities, type of feeding and initiation of sleep and waking up. |
|  | Time patterns in parent‐child interactions in a trobriand village (Papua New Guinea)  Siegmund et al.,  1994 (Siegmund et al. 1994) | Observe parent-infant interactions in a traditionally living society over a 7-day period using a microelectronic actometer. | 4 families, mother, father and children (overall n=39). | Mother, father and infant synchrony. | Infants were 1,2,5, and 11 months. | Inhabitants of Trobriand Islands, society with no access to electricity. | Observational. | For 7 continuous days they wore an activity monitor (actometer) which registered data every two minutes. |
| Sleep Synchrony | | | | | | | | |
|  | Concordance in parent-child and sibling actigraphy-measured sleep: Evidence among early adolescent twins and primary caregivers  Sasser et al.  2023 (Sasser et al. 2023) | To investigate daily and average concordance in parent-youth and sibling actigraphy measured sleep. | 516 twin siblings and their primary caregivers. | Parent, child and sibling synchrony. | Mean age 10.47 years. | 393 families were part of the 10-year study wave of the ongoing Arizona Twin Project. | Cross sectional. | Wrist-based accelerometery for 7 consecutive nights.  Primary caregivers provided information on self and twin wake times and bedtimes through assessment tables to cross reference actigraphy measures. |
|  | In or out of sync? Concordance between parent and adolescent sleep varies by family context.    Sasser and Oshri    2023 (Sasser and Oshri 2023) | To examine the daily and average concordance between parent and adolescent sleep and to explore adverse parenting and family functioning as potential moderators. | 124 adolescents and their parents (93% mothers). | Parent-child sleep concordance. | Children were aged 12-14 years old (mean age 12. 90). | Children were required to be 12-14 years old, fluent in English, and able to read and answer questions in the survey. | Cross sectional. | Actigraphy was used to measure sleep duration, sleep efficiency and sleep midpoint time over 1 week (average 6.85 nights of sleep recorded).  The Parent-Child Conflict Tactic Scale was used to measure adverse parenting.  The Family Adaptability and Cohesion Evaluation Scale was used to determine family functioning. |
|  | Actigraphy-measured sleep concordance, night-wakings, intraindividual sleep variability in parents and their children—Associations with childhood sleep disturbances    Varma et al.    2022 (Varma et al. 2022) | To determine sleep-wake concordance and to identify temporal associations between children’s night-wakings and parent’s night-wakings. | 20 parents and children.  Children with (n=11) and without (n=9) sleep disturbance. | Parent and child sleep concordance. | Children were aged 2-12 years old. | Primary caregiving parents, fluent in English and aged 18 years and above with children aged 2-12 years old.  Individuals were excluded if the parent was receiving treatment for a sleep disorder other than insomnia, if the parent was breastfeeding or had another child under the age of 2 years old, the parent was engaged in regular shift work or the parent was co-sleeping with the child. | Pilot study, case-control. | Actigraphy over 14 nights.  Pittsburg Sleep Quality Index  Children’s Sleep Habits Questionnaire.  A sleep diary (completed for parents and children) was used to measure subjective perceptions of bedtime, waketime, wakings and total sleep time over the 14 days. |
|  | A longitudinal study of the links between maternal and infant nocturnal wakefulness.    Tikotzky et al.    2021 (Tikotzky et al. 2021) | To examine the longitudinal links between maternal and infant nocturnal wakefulness and to assess whether the strength of mothers' links differs as a function of sleep assessment method and sleeping arrangements. | 216 couples. | Mother-infant synchrony. | Measures were taken when infants were 3 (n=191), 6 (n=178), 12 (n=155) and 18 (n=135) months postpartum. | Inclusion criteria included 2-parent families with a singleton pregnancy and a healthy infant born at term (>37 weeks).  Post hoc exclusion criteria included infant or maternal breathing related problems. | Longitudinal. | Actigraphy and sleep diaries over 5 nights - mothers were asked to record their own and their infant’s sleep schedules and night-wakings in the morning following each assessment night.   The Brief Infant Sleep Questionnaire (BISQ) was used to assess sleeping arrangements. |
|  | The role of parental circadian preference in the onset of sleep difficulties in early childhood    Morales-Munoz et al.    2019 (Morales-Muñoz et al. 2019) | To investigate the association between parental circadian preference and sleep in childhood. | In total, 1220 mothers, 1116 fathers, 993 infants at three months, 990 infants at eight months, 958 children at 18 months, and 777 children at 24 months were analysed. | Parent and child synchrony. | Measures were taken when children were 3, 8, 18 and 24-months. | Healthy infants.  Individuals with any medical illness and/or reported condition at any time point were excluded. | Longitudinal. | A shortened version of the Horne-Ostberg Morningness-Eveningness Questionnaire (MEQ) was used for parents.  The Brief Infant Sleep Questionnaire (BISQ) and the Infant Sleep Questionnaire (ISQ) was used for infants at each time point. |
|  | Sleep: population epidemiology and concordance in Australian children aged 11–12 years and their parents    Matricciani et al.    2019 (Matricciani et al. 2019) | To describe objectively measured sleep characteristics in children aged 11–12 years and in parents and to examine intergenerational concordance of sleep characteristics. | 1261 children, 1358 parents. A total of 1077 biological parent–child pairs. | Parent-child synchrony. | Children had a mean age of 12 years and parents had a mean age of 43.8 years. | Children aged 11-12 years. | Cross-sectional study. | Accelerometery over 8 days.  Participants also completed a self-report record to record bedtime and wake times and any time they removed the accelerometer and the reasons for removing it. |
|  | The Transition of Sleep Behaviours in Twin Infants and Their Mothers in Early Infancy    Kondo and Takada.  2018 (Kondo and Takada 2018) | To clarify the sleep behaviours of twin infants and their mothers by using actigraphy (four measurement periods at approximately 4-to-6-week intervals) and to evaluate the relationship between mother and infant sleep behaviours.  . | 5 twin pairs and their mothers (first time mothers) | Mother-infant synchrony.  Twin-twin synchrony. | Infants were a corrected age of 3-6 weeks, 8-11 weeks, 13-15 weeks, 17-20 weeks.  Maternal age (mean) 32.8 years old. | No neurological or developmental problems in infants, primipara mothers, and mothers with male partners. | Longitudinal. | Infants’ and mothers’ sleep behaviours were recorded for seven consecutive days at four timepoints using an actigraph (1-minute intervals)  Sleep diaries were also recorded with information on the times when the actigraph was removed, when infants went to bed and left bed, when infants had been fed, and when infants had their diapers changed.  Mothers completed the Edinburgh Postnatal Depression Scale (EPDS) at the beginning of the study. |
|  | Within-Family Relations in Objective Sleep Duration, Quality, and Schedule    Kouros and El-Sheikh    2017 (Kouros and El-Sheikh 2017) | To examine within-family relations between mothers’, fathers’, and children’s objectively assessed sleep. | 163 two parent families - children and their parents. | Parent-infant synchrony. | On average, children were 10.45 years old.    Mothers and fathers mean age was 36.93 and 40.02, respectively. | All families lived together and slept in the same house.  Individuals were excluded if they were from a single-parent household, if one or both parents were a shift worker and families that did not have useable sleep data for at least two family members. | Cross-sectional. | Actigraphy and sleep diaries (for each parent and child, mother completed sleep diaries for the child) over 7 consecutive nights. |
|  | Daily Concordance Between Parent and Adolescent Sleep Habits    Fuligni et al.    2015 (Fuligni et al. 2015) | To assess the daily concordance between parent and adolescent daily sleep habits, how that concordance compares to other predictors of sleep, and whether the degree of concordance varies across families. | 421 adolescents and their primary caregivers (83% mothers, 13% fathers and 4% other family members). | Caregiver-child sleep concordance. | The mean age of children was 15.03 years old and the mean age of primary caregivers was 41.93 years old. | Adolescents with Mexican backgrounds and their primary caregivers. | Cross-sectional | Measures were taken at two 14 days periods across two years.  Interview and questionnaire measures in both waves of the study assessed parent education and household size and interpersonal support and conflict.  Daily checklists across 14 days in both waves of the study. Checklists assessed sleep, wake and bedtimes, adolescent study time and parent work, adolescent and parent demands.  Follow up at 1 year. |
|  | Genetic and Environmental Contributions to Sleep-Wake Behavior in 12-Year Old Twins  Sletten et al  2013 (Sletten et al. 2013) | To examine the role of genetic and environmental factors on sleep behavior in 12-year-old twins matched for family environment. | 132 twins (including 25 monozygotic twins and 41 dizygotic twins). | Twin-twin synchrony. | 12 years old. | Participants were a subsample of the Brisbane Adolescent Twin Study. | Population based twin cohort. | Sleep diary and actigraphy for 2 weeks. |
|  | In sync with the family: children and partners influence the sleep-wake circadian rhythm and social habits of women.    Leonhard and Randler.    2009 (Leonhard and Randler 2009) | The aims of this study were to assess the influence of children and partners on the social habits of women at different stages of their lives and to assess their effect on synchrony within the family. | 179 women: women without children and non-pregnant (n=49), pregnant women without children (n=35), pregnant women with children (n=26), and non-pregnant mothers (n=69). | Mother, partner, and child synchrony. | Mean age of women without children and non-pregnant: 23.20 years old.  Mean age of women without children and pregnant: 29.6 years old  Mean age of women with children and non-pregnant: 33.26 years old  Mean age of women with children and pregnant: 33.42 years old.  Mean age of first child was approx. 3.3 years old. | Without children and non-pregnant, without children and pregnant, with children and non-pregnant, with children and pregnant  All mothers in the study lived with a partner. | Cross-sectional. | Composite Scale of Morningness.  Questionnaire on Daily Patterns Derived from the Social Rhythm Metric – 5 (SRM-5).  Subjective sleep-onset latency was assessed by a categorical scale.  Weekend oversleep was calculated according to Wolfson & Carskadon (1998). It measures the difference between sleep length weekdays and weekends. The difference between both is termed weekend oversleep.  The women were asked for rise and bedtimes on weekdays and on the weekend to calculate the midpoint of sleep on free days. |
|  | Relationship Between Child Sleep Disturbances and Maternal Sleep, Mood, and Parenting Stress: A Pilot Study  Meltzer and Mindell  2007 (Meltzer and Mindell 2007) | To examine the relationship between child sleep quality and maternal sleep quality, to determine whether maternal functioning is related to child sleep disruptions. | 47 mothers. | Mother and child sleep. | Mothers were 30-50 years.  Children’s ages ranged from 3-14.4 years. | Mothers were excluded if they had a diagnosed sleep disorder, their child had a diagnosed sleep disorder, chronic illness, or developmental delay, the family did not have access to a telephone, the mother was unable to complete the questionnaires in English | Pilot study. | Children’s Sleep Habits Questionnaire.  24-hour Sleep Patterns Inventory.  Centre for Epidemiological Studies – Depression Scale.  Overload questionnaire.  Parental distress measure.  Iowa Fatigue Scale.  Standford Sleepiness Scale. |
|  | Family synchronizers: Predictors of sleep–wake rhythm for Japanese first-time mothers.    Yamazaki    2007 (Yamazaki 2007) | To describe the association between the regularity of sleep patterns for first-time Japanese mothers in the early postpartum period and the sleep and wake activity of their infants and partners. | 101 couples. | Mother, partner, child synchrony. | Data was obtained at 32-36 weeks pregnancy (this provided baseline rhythms of the couple) and 4-5 weeks post birth.  Mothers were 29.5 +/- 3.8 years, fathers were 31.6 +/- 4. 9 years. | Inclusion criteria were no pre-existing chronic psychiatric illness and no obstetrical complications, couples working day shifts if they are working; and infants not requiring a revisit or follow up at 1-month postpartum examination. | Longitudinal. | Sleep-wake log for 7 days: this assessed sleep-wake time and sleep-wake rhythm strength of parents as well as recording infant sleeping and caregiving activities.  At the postpartum period the mothers also recorded the infant’s sleep–wake activity every 15 min in the log.  A social rhythm metric for 7 days: assessed regularity of social daily rhythms.  Morningness-eveningness questionnaire. |
|  | Infant sleep and feeding pattern: Effects on maternal sleep    Thomas & Foreman.    2005 (Thomas and Foreman 2005) | The aims were to describe maternal and infant sleep pattern, examine the influence of feeding on the sleep-wake pattern of both mothers and infants, and explore the effects of infant and maternal factors on maternal sleep. | 37 mother-infant pairs. | Mother-infant synchrony | Infants were 4-10 weeks postnatal age. | Criteria for maternal participation included the following:  age 18 to 40 years, primiparous, vaginal birth, and absence of antepartum or postpartum health problems.  Infants were 38 to 42 weeks’ gestation, 4 to 10 weeks’ postnatal age, singleton birth, with no postnatal complications.  Mothers were screened for postpartum depression prior to taking part using the Edinburgh Postnatal Depression Scale. | Cross-sectional. | Sleep-Activity Record: a 24-hour sleep-wake diary divided into 15-minute intervals. Mothers entered codes to indicate mother-infant activity.  Only recorded over one 24-hour period. |
|  | Similarities and Differences in Sleep-Wake Patterns Among Adults and Their Children    Gau and Merikangas.    2004 (Gau and Merikangas 2004) | To determine associations and differences in sleep-wake patterns among children and their parents and to explore the correlates for sleep-wake patterns among adults. | 1479 parents and 1335 child participants (1335 dyads in total).  195 father-son dyads.  485 mother-son dyads.  162 father-daughter dyads.  493 mother-daughter dyads. | Parent-child synchrony. | Children were aged 10-16 years old, mean age of 12 years old. | Excluded if more than half of the items on the adult M/E scale were in complete.  Only parents who had regular daytime and work schedules and their children were included in the analysis of parent-child sleep-wake pattern associations and differences. | Cross-sectional study. | Sleep habits questionnaire (SCQH).  Morning-eveningness questionnaire.  Mood scale. |
|  | The development of infants’ circadian rest–activity rhythm and mothers’ rhythm  Nishihara et al.  2002 (Nishihara et al. 2002) | To examine the development of the circadian sleep-wake cycle in infants and changes in the sleep wake cycle in their mothers in the postpartum period. | 11 primipara and their infants.  Eight infants were breast fed and three were formula fed. | Mother-infant synchrony. | Mean age of mothers was 28.8 years.  Infants were recorded during their 3^rd^, 6^th^, 9^th^ and 12^th^ weeks. | All infants were vaginally born and full-term. | Longitudinal. | Actigraphic recordings for the infants and their mothers were made over 3–5 continuous days during the 3rd, 6th, 9th and 12th weeks after birth.  The mother kept sleep and feeding logs each day and noted the times they put and took off the Actiwatches each day. |
|  | Mothers’ wakefulness at night in the post-partum period is related to their infants’ circadian sleep–wake rhythm.  Nishihara,  2000 (Nishihara et al. 2000) | To investigate the relationship between a post-partum mother’s wakefulness at night and her infant’s circadian sleep–wake rhythm. | 7 mothers and infants. | Mother-child synchrony. | Infants were recorded in their 3^rd^, 6^th^, 9^th^ and 12^th^ weeks.  Mean age of the mother’s was 27.6 years. | All infants were full-term.  There were no shift workers among the parents. | Longitudinal. | Actigraphy recordings over 3-5 continuous days during weeks 3, 6, 9 and 12 using an Actiwatch.  3 days during the 3^rd^ week and 5 days during the 6^th^, 9^th^ and 12^th^ weeks |
|  | Activity Monitoring of the Inhabitants in Tauwema, a Traditional Melanesian Village: Rest/Activity Behaviour of Trobriand Islanders (Papua New Guinea)  Siegmund et al.  1998 (Siegmund et al. 1998) | To examine sleep-wake patterns and development of biological rhythms in a traditional culture. | 7 families – 39 individuals in total. | Mother, father and child. | Infants aged between 1 and 5 months and children aged between 11 months and 6 years. | Families or people living in one household were preferentially chosen.  Traditional culture with no artificial light | Cross-sectional. | Families were recorded continuously for 7 days using microelectronic actometers that register locomotor activity. |
|  | Sleep and arousal, synchrony and independence, among mothers and infants sleeping apart and together (same bed): an experiment in evolutionary medicine  McKenna and Mosko  1994 (McKenna and Mosko 1994) | To summarise the effect of same-bed co-sleeping on sleep and arousal patterns of mother-infant pairs.  Describes two pilot studies on co-sleeping mothers and infants. | Study one: consisted of 5 healthy mother-infant pairs who co-slept one night in a sleep laboratory.  Study two: three additional mother infant pairs were studied for 3 consecutive nights. | Mother-infant synchrony. | Infants were full-term aged between 2-5 months. | Mothers in both studies had normal pregnancies and deliveries, and a medical examination of infants one week prior to the study showed they all had normal developmental histories. | Two pilot studies. | Study one: polysomnographic recording taken in a laboratory over one night.  Study two: recordings were taken over three nights. Pairs slept apart in adjacent rooms over the first two nights and side-by-side on the same bed for the third night.  Sleep pattern (EEG, electrooculogram and chin electromyogram), respiration (chest wall movement and sometimes oronasal airflow) and EKG were monitored non-invasively each night. All mother and infant channels recorded simultaneously on a single polygraph.  In both studies mothers completed a questionnaire upon wakening. |

**Supplementary Table 3**

*Results of individual studies that included families with autistic children.*

|  | Citation: Title, author(s), year of publication | Study design | Statistics used to parameterise synchronisation | Comparison/control | Outcome/results in relation to the synchrony of circadian rhythms within the family |
| --- | --- | --- | --- | --- | --- |
| Sleep Synchrony | | | | | |
|  | Familial sleep and autism spectrum disorder: a pilot actigraphy study of sleep quality, quality of life and psychological distress  Leader et al  2021 (Leader et al. 2022) | Pilot study. | Pearson’s correlations. | No. | There was no relationship between child actigraphy variables and parent actigraphy variables (bedtime, get up time, time in bed, total sleep time, onset latency, sleep efficiency, wake after sleep onset, number of wake intervals). |
|  | Concordance of Mother/Child Sleep Patterns Using Actigraphy: Preliminary Findings  Goldman et al.  2014 (Goldman et al. 2014) | Pilot study. | Ranked two-sample comparison and correlation. | Yes: autism-good sleepers (n=6), autism-poor sleepers (n=5) and non-autistic children (classified as good sleepers). | Positive associations were found between autism-good sleeper mothers and their children for bedtime and total sleep time.  The autism-poor sleeper mothers and their children showed associations for sleep efficiency, fragmentation, wake after sleep onset and the percentage wake bouts.  Mothers of non-autistic children and their children showed associations for total sleep time.  When all groups were combined there was significant associations between mothers and their children in terms of bedtime, total sleep time and sleep fragmentation. |
|  | Relationship between children's sleep and mental health in mothers of children with and without ASD  Hodge et al.  2013 (Hodge et al. 2013) | Case-control. | Path analysis. | Yes –90 non-autistic children who were age-, gender-, and ethnicity-matched to autistic participants. | Children’s sleep significantly predicted maternal mental health, maternal sleep and maternal stress.  Maternal sleep and maternal stress predicted maternal mental health in mothers with autistic children and mothers with non-autistic children.  Stronger relationship between children’s sleep and maternal stress for mothers of non-autistic children. |
|  | Sleep problems among Taiwanese children with ASD, their siblings and typically developing children  Chou et al.  2012 (Chou et al. 2012) | Case-control. | Linear mixed models. | Yes. 110 autistic children with 110 age-, sex-, and parental education-matched non-autistic children and 125 non-autistic siblings. | Autistic children tended to have early insomnia, middle insomnia, sleep–wake schedule disorders and daytime inadvertent napping compared to unaffected siblings and non-autistic controls.  Unaffected siblings were more likely to have early insomnia, sleep-talking and nightmares compared to non-autistic controls.  Autistic children and non-autistic siblings tended to wake up later on weekdays than non-autistic controls. Autistic children woke earlier on weekends compared to non-autistic siblings and non-autistic controls.  There was no significant difference in bedtimes or sleep duration during weekdays or weekends between the three groups.  In terms of their sleep patterns between weekdays and weekends, both autistic children and non-autistic siblings had a smaller difference in bedtime and rise time than non-autistic controls.  Only autistic children had a significantly smaller weekday-weekend difference in sleep duration than non-autistic controls. |
|  | Factors associated with depressive symptoms in parents of children with autism spectrum disorders  Meltzer.  2011 (Meltzer 2011) | Case-control. | Pearson’s correlations  Hierarchical multiple regression analyses. | Yes – TD group: 17 non-autistic children, 17 mothers, 11 fathers. | Autistic children slept for 30 minutes less on average than non-autistic children but there was no significant difference between the two groups in terms of sleep quality.  Significant associations were found for child objective sleep quantity and maternal reports of child sleep disruptions meaning that children with shorter sleep time by actigraphy have more maternal reported sleep disruptions.  No association was found for child objective sleep quality and paternal reports of child sleep disruptions.  Significant associations were found between child sleep disturbances and parent sleep quality; suggesting that parents who report more sleep disturbances in their children also report poorer sleep quality for themselves. |
|  | Sleep problems of parents of typically developing children and parents of children with ASD  Lopez-Wagner et al.  2008 (Lopez-Wagner et al. 2008) | Cross-sectional correlation. | Correlations between children sleep habits questionnaire scores and global Pittsburgh sleep quality index score.  Multivariate analysis of variance | Yes - 168 parents and children. 4-15 years old in the community group. | Parents of autistic children reported that they experienced more sleep problems than did parents of non-autistic children.  Significant correlations were found between parents’ reports of their children’s sleep problems and their own sleep difficulties for both the autism and community groups (these correlations were not significantly different from one another).  When children’s sleep problems were more severe, as in the autism group, parents reported that their own sleep problems were more affected.  For autistic children, sleep problems and not their autistic symptoms were related to parents’ reported sleep difficulties. |

**Supplementary Table 4**

*Results of individual studies that included only families with non-autistic children.*

|  | Citation | Study design | Statistics used to parameterise synchronisation | Comparison/  control | Outcome/results in relation to the synchrony of circadian rhythms within the family |
| --- | --- | --- | --- | --- | --- |
| Biomarker Synchrony | | | | | |
|  | Mother-child adrenocortical synchrony; Moderation by dyadic relational behavior  Pratt et al.  2017 (Pratt et al. 2017) | Cross-sectional. | Multilevel modelling was used to test mother-child diurnal cortisol synchrony.  t-tests and correlations were also used. | Yes – 69 non-depressed controls. | Maternal cortisol across the three measurements was significant in predicting child cortisol beyond the effect of diurnal variation.  Maternal depression was not found to moderate cortisol linkage.  Mother-child dyadic reciprocity showed a significant negative moderating effect, indicating that the more dyadic reciprocity was observed in the mother-child interaction the less mother and child's diurnal cortisol were coupled.  The greater the child's cortisol secretion throughout the day, the stronger the linkage between maternal and child's diurnal CT. |
|  | The effects of SES on infant and maternal diurnal salivary cortisol output  Clearfield et al.  2014 (Clearfield et al. 2014) | Cross-sectional. | Correlations and Mixed model Analysis of Variance (ANOVA) | Comparison between 16 high-SES mothers and infants and 16 low-SES mothers and infants. | Low SES infants had overall higher cortisol levels than high SES infants.  There were correlations between maternal and infant cortisol levels separately for each time of day and by SES:  High SES dyads were marginally correlated in the morning, significantly correlated in the evening, and not correlated in the afternoon.  Low SES dyads were not significantly correlated at any time of the day and all correlations were negative – they were increasingly negatively correlated in the afternoon and evening, suggesting that the build-up of daily stress is related to more divergence.  Overall, more synchrony was evident in high SES dyads than in low SES dyads. |
|  | Exploring patterns in cortisol synchrony among anxious and nonanxious mother and child dyads: a preliminary study  Williams et al.  2013 (Williams et al. 2013) | Pilot/exploratory study.  Cross-sectional. | Bivariate correlations, multiple regression equations, actor-partner independence measure, hierarchical linear modelling. | No. | There was a significant association between basal cortisol of children and basal cortisol of mothers. |
|  | Comparisons between salivary cortisol levels in six-months-olds and their parents  Stenius et al.  2008 (Stenius et al. 2008) | Cross-sectional. | ANOVA. | No. | Strong correlations between cortisol levels in mother and infant samples in the morning, afternoon, and evening.  Weaker correlations in cortisol levels between infant and father’s samples and only in the afternoon and evening samples. In the morning father and infant cortisol levels were not associated with one another.  There was a strong relationship between waking up/bedtime-difference in mother-child and a weaker relationship between waking up/bedtime-difference in father-child. |
|  | The circadian rhythm of tryptophan in breast milk affects the rhythms of 6-sulfatoxymelatonin and sleep in newborn  Cubero et al.  2005 (Cubero et al., 2005) | Case-control. | Kruskal-Wallis test and matched pairs signed rank tests. | Yes. Healthy breastfed infants and healthy formula fed infants aged 12 weeks. | The circadian rhythm of 6-sulfatoxymelatonin in exclusively breastfed infants was influenced by the rhythm of tryptophan in their mother's milk. |
| Activity synchrony | | | | | |
|  | The influence of feeding method on a mother's circadian rhythm and on the development of her infant's circadian rest-activity rhythm  Kikuchi et al.  2020 (Kikuchi et al. 2020) | Longitudinal. | Autocorrelograms. | No. | The circadian rest-activity rhythm of the breastfed infants had started at the 2nd-3rd week, with it becoming clearly present in the 6^th^ week, while that of the mixed-fed infants was delayed but was established by the 12th week.  The regularity of the circadian rhythm of the breastfed infants was definite in comparison with that of the mixed-fed infants.  Both groups of mothers kept their own circadian rest-activity rhythm from the 2nd-3rd week to the 12th week. The circadian rhythms of breastfeeding mothers were more regular than that of mixed feeding mothers.  The authors conclude that breastfeeding contributes to infant's obtaining circadian rest-activity rhythm. |
|  | Accelerometery-Derived Physical Activity Correlations Between Parents and Their Fourth-Grade Child Are Specific to Time of Day and Activity Level.  Strutz et al.  2018 (Strutz et al. 2018) | Cross-sectional. | Bivariate and partial correlations. | No. | Weak to moderate correlations were found between parent and child moderate-vigorous physical activity levels across the seven days.  Moderate-vigorous physical activity was significantly correlated in all dyads before school, after school, during evening periods and on weekend days.  Children’s moderate-vigorous physical activity level accumulation did not differ between children with more or less physically active parents.  Parent’s moderate-vigorous physical activity levels did differ based on their children’s activity levels during the evening – parents with more active children were more active than parents whose children were less active. |
|  | Application of Empirical Mode Decomposition to Mother and Infant Physical Activity: Synchronisation of Circadian Rhythms is Associated with Maternal Mental Health  Shimizu et al*.*  2018 (Shimizu et al. 2018) | Cross-sectional | Bivariate empirical mode decomposition  Multi-level modelling | No. | There was a significant association between maternal mental health and desynchronisation of mother–infant circadian rhythms of physical activity.  Diurnal fatigue and depressive mood scores were significantly and positively correlated with an increase in mother-infant phase differences. |
|  | Light and maternal influence in the entrainment of activity circadian rhythm in infants 4–12 weeks of age  Thomas et al.  2016 (Thomas et al. 2016) | Longitudinal cross-sectional study. | Cosinor and nonparametric circadian rhythm analyses.  Partial correlations. | No. | All maternal and infant circadian measures for light were highly correlated.  The correlation between infant activity rhythm and light increased between 8-12 weeks.  The partial correlations between infant activity and light rhythm timing, amplitude, 24-h fit, and rhythm centre were significant were significant at 12 weeks.  When maternal light was controlled for, there was significant correlation between maternal and infant activity rhythms.  Both light and maternal activity may offer avenues for shaping infant activity rhythm during early infancy. |
|  | Mother–infant circadian rhythm: Development of individual patterns and dyadic synchrony  Thomas et al.  2014 (Thomas et al. 2014) | Longitudinal | Cosinor  Non-parametric analysis. | No. | Mothers experienced early disruption of circadian rhythm, with re-establishment of rhythm over time.  Infants demonstrated a developmental trajectory of circadian pattern with increasing mesor, magnitude, amplitude, midpoint of lowest 5 (L5), interdaily stability, and intradaily variability.  Infants increasingly phase advanced relative to mother over study duration. Evidence of mother-infant synchrony in increasing correspondence of acrophase. |
|  | Relationship Between Infant and Mother Circadian Rest-Activity Rhythm Pre- and Postpartum, in Comparison to an Infant With Free-Running Rhythm  Nishihara et al.  2012 (Nishihara et al. 2012) | Longitudinal | Autocorrelogram, t tests , one-way, repeated-measures ANOVA | No. | Infants circadian rest-activity rhythms were present at 2 weeks and amplitude increased up to 12 weeks. The infants rest-activity rhythm was established by the 12^th^ week.  Amplitude of mother's autocorrelogram at 24h decreased after birth, because of increased night-time waking for infant care. The mother’s circadian rhythm became more regular from the 2^nd^-12^th^ weeks but did not return to pre-partum levels. This shows that mothers had to alter their circadian rest-activity rhythm to care for their infants.  One infant showed a free-running pattern of activity. This infant’s mother had peaks in her circadian rhythm and showed a split rhythm from the 10^th^-11^th^ weeks (20- and 26-hours peaks). These peaks were also seen in the mother’s late pregnancy autocorrelograms. At 12 weeks, circadian rest-activity rhythm peaks were observed for the mother and infant and the infants peak appeared to be earlier than the mothers. It appeared that the infant led the mother’s rhythm to be free running. |
|  | Development of synchrony between activity patterns of mother-infant pair from 4 to 18 months after birth  Doi et al.  2011 (Doi et al. 2011) | Cross-sectional. | The strength of mother–infant synchrony was estimated by cross-correlation analysis.  Multi-variable regression analysis was also conducted. | No. | The main finding is that cross-correlation of activity patterns between mother and infant increased from 4 to 18 months after birth, indicating a consolidation of mother–infant synchrony during this period.  Results also suggest that synchrony strength is uniquely related to age in days of infants. |
|  | Mother-infant activity synchrony as a correlate of the emergence of circadian rhythm  Tsai et al*.*  2011 (Tsai et al. 2011) | Cross-sectional study. | Cosinor analysis, pearson correlation and regression analysis. | No. | 2–10-week-old infants have beginning circadian rhythms and the acrophase of their activity shows an adult-like response.  There was a strong within dyad correlation for activity as well as a strong correlation between mother and infant circadian activity patterns. This suggests that infants are behaviourally entrained to the 24-hour day by their mothers.  Within dyad correlation of activity was associated with greater amplitude and the robustness of the infant rhythm. |
|  | Development of the 24-h rest-activity pattern in human infants  Jenni et al.  2006 (Jenni et al. 2006) | Longitudinal | Periodogram analysis | No. | Ultradian pattern of rest-activity was predominate in the new-born period. The infants gradually developed a rhythm of 24-hours – with a night preference for rest and a day preference for activity.  Within the first few weeks after birth mothers had increased nighttime activity and daytime napping.  There was large variation between infants in daytime rest behaviour. |
|  | Circadian and Ultradian Time Patterns in Human Behavior: Part 2: Social Synchronisation During the Development of the Infant's Diurnal Activity-Rest Pattern  Wulff et al.  2001 (Wulff et al. 2001) | Longitudinal | Fast Fourier transformation.  Cross correlation. | Yes – synchronisation of a Melanesian family was compared to understand the differences in social synchronisation between cultures. | Concordant ultradian frequencies in mother infant pairs at 1, 2 and 4 months.  Increases in the synchronisation of parental activity were found from prenatal to postnatal and from the first to the second month for mothers and infants.  Mother-infant pairs were more synchronised than father-infant pairs.  Good correlation between mother-infant activity was associated with rapid development of entrained circadian rhythms in infants.  Circadian pattern emerged during first few weeks . |
|  | Circadian and Ultradian Time Patterns in Human Behaviour: Part 1: Activity Monitoring of Families from Prepartum to Postpartum    Wulff & Siegmund  2000 (Wulff and Siegmund 2000) | Longitudinal. | ANOVA, non-parametric tests. | Yes – data was collected for seven non-pregnant women and used as a control. | Activity at night increased from the prenatal to the postnatal period in mothers and fathers.  All mothers’ nocturnal activity coincided with that of their infants’ activity, the fathers were affected by indirect disturbances.  The majority of the mother-infant pairs showed a high correlation of concurrent onset of daytime activity.  Mothers had lower amplitude circadian rhythms after birth.  All infants showed a predominant circadian rhythm between day 8 and 19 after birth. |
|  | Time patterns in parent‐child interactions in a trobriand village (Papua New Guinea)  Siegmund et al.  1994 (Siegmund et al. 1994) | Observational. | Descriptive. | No. | Synchronisation was evident between infants and mothers during the day and night, but no father-infant synchronisation was found. There was a difference in infant and mother’s rhythmicity depending on the infant’s age.  In older infants, 5-11 months, there was no synchronisation of parents’ or siblings’ movements in relation to the infant’s nocturnal activities. |
| Sleep Synchrony |  |  |  |  |  |
|  | Concordance in parent-child and sibling actigraphy-measured sleep: Evidence among early adolescent twins and primary caregivers  Sasser et al.  2023 (Sasser et al. 2023) | Cross-sectional. | Multi-level models. | No. | There was significant daily and average sleep concordance between parent and youth sleep duration and sleep mid-point time.  Parent and child sleep efficiency were not significantly related to one another at the daily or average level.  There was significant average but not daily concordance between parent and child sleep latency.  There was significant concordance between twins’ sleep duration, sleep efficiency, sleep midpoint and sleep latency at a daily and average level.  Siblings who slept in the same room had greater concordance of sleep measures. |
|  | In or out of sync? Concordance between parent and adolescent  sleep varies by family context.  Sasser and Oshri  2023 (Sasser and Oshri 2023) | Prospective observational. | Multi-level models. | No. | At a daily level, parent and child sleep duration and sleep midpoint were significantly associated. There were no significant daily associations between parent and child sleep efficiency.  On average, parent and child sleep midpoint was significantly associated but parent and child sleep efficiency and sleep duration were not significantly associated.  Family flexibility was associated with higher concordance in sleep duration and midpoint.  Adverse parenting was associated with discordance in sleep duration and efficiency. |
|  | Actigraphy-measured sleep concordance, night-wakings, intraindividual sleep variability in parents and their children—Associations with childhood sleep disturbances  Varma et al.  2022 (Varma et al. 2022) | Pilot study, case-control. | Concordance analysis, defined as the percentage of actigraphic epochs where both parents and children were in a sleeping or waking state.  Blip analysis, where a blip was defined as the starting five minutes of night-time awakenings.  The concordance and temporal order of blips between parents and children were categorised.  Blip “hit rate” was the proportion coincidence of blips between subjects or timepoints. | Children with and without sleep disruption and their parents. | Sleep concordance between parent-child ranged between 58-89% (mean 70.6%). The percentage of times the parent was awake during their child’s waking was significantly higher in comparison to the percentage of times the child was awake during their parent’s wakings.  33% of parent night-wakings occurred within 10 min of their child's waking in comparison to < 11% of children waking up within 10 min of their parent's night-waking.  Parents of children with sleep disturbances displayed significantly poorer sleep quality, higher wake after sleep onset (WASO), and greater variability in sleep duration and bedtime in comparison to parents of children with no sleep difficulties.  Parent reported child sleep difficulties were corroborated on the actigraphy of the children. |
|  | A longitudinal study of the links between maternal and infant nocturnal wakefulness  Tikotzky et al.  2021 (Tikotzky et al. 2021) | Longitudinal. | Pearson correlation and multi-level models. | No. | There were significant associations between mother and infant sleep at all assessment points (in terms of the amount of night wakenings and time spent awake). The strength of this association declined over time.  These associations were stronger for maternal reports than actigraphy measures.  There was no significant difference in the correlation between sleep measures and sleeping arrangements (room-sharing vs solitary-sleeping families). |
|  | The role of parental circadian preference in the onset of sleep difficulties in early childhood  Morales-Munoz et al.  2019 (Morales-Muñoz et al. 2019) | Longitudinal. | Regression analysis. | No. | Maternal circadian preference affects the development of infant’s circadian rhythm - maternal eveningness preference is associated with slower circadian rhythm development in infants at three, eight, 18 and 24 months.  Maternal eveningness was associated with short sleep time duration during the daytime at 8 months during the night at 3 and 8 months, with long sleep-onset latency at 3, 18 and 24 months, with late bedtime at 3, 8, 18 months and with parent-reported sleep difficulties at 8 and 24 months.  There was no link between paternal circadian preference and children’s sleep at any time point. |
|  | Sleep: population epidemiology and concordance in Australian children aged 11–12 years and their parents  Matricciani et al.  2019 (Matricciani et al. 2019) | Cross-sectional study. | Pearsons correlation coefficients and regression coefficients. | No. | Parent-child concordance was evident for all measures including sleep duration, sleep onset, sleep offset, day to day variability in duration and efficiency.  Concordance was strongest for sleep onset and offset and in mother-child pairs (notably, mother-child pairs made up the majority of the participants). |
|  | The Transition of Sleep Behaviours in Twin Infants and Their Mothers in Early Infancy  Kondo and Takada.  2018 (Kondo and Takada 2018) | Longitudinal. | Kruskal-Wallis one-way analysis of variance and Wilcoxon’s rank test.  Spearman correlation coefficients. | No. | Twin infants sleep patterns changed from 3-6 weeks to 8-11 weeks – wake duration decreased, and sleep duration increased. The proportion of time with both twins asleep significantly increased.  Maternal sleep duration during both infants sleeping was significantly correlated with corrected age. Maternal sleep duration increased with the synchronisation of sleep behaviours between twin infants. |
|  | Within-Family Relations in Objective Sleep Duration, Quality, and Schedule  Kourous and El-Sheikh.  2017 (Kouros and El-Sheikh 2017) | Cross-sectional. | Multilevel models. | No. | Children’s sleep duration and sleep quality were related to their mother’s sleep on the same night and to wake times the next morning.  Mother’s sleep was influenced by child and partners sleep on the same night.  Father’s sleep was predicted by their partners sleep but not by their children’s sleep. |
|  | Daily Concordance Between Parent and Adolescent Sleep Habits  Fuligni et al.  2015 (Fuligni et al. 2015) | Prospective observational. | Multilevel models. | Within family and between family comparisons. | There was significant concordance between parent and child daily sleep time. This concordance was equally strong for both parents and adolescents and appeared to be attributed to concordance between both bed and wake times for parents and their children.  Concordance between parent and child sleep remained even after controlling for other experiences.  Concordance was strongest among larger families and those with more support parent-adolescent relationships. |
|  | Genetic and Environmental Contributions to Sleep-Wake Behavior in 12-Year Old Twins  Sletten et al  2013 (Sletten et al. 2013) | Population based twin cohort. | Correlations. | Sleep was compared within twin pairs. | There were correlations in sleep habits between both monozygotic and dizygotic twins.  There were greater correlations in sleep habits (sleep onset, sleep efficiency and sleep fragmentation) in monozygotic than in dizygotic twins.  There was also a trend of greater correlations in total sleep time and start and end of sleep time in monozygotic than in dizygotic twins, but this was not significant.  Sleep phenotypes between monozygotic twins were similar. |
|  | In sync with the family: children and partners influence the sleep-wake circadian rhythm and social habits of women.  Leonhard and Randler.  2009 (Leonhard and Randler 2009) | Cross-sectional. | Several different types of analyses were used: correlations, regression analysis, partial correlation, and general linear models. | Comparison between women without children and non-pregnant (n=49), pregnant women without children (n=35), pregnant women with children (n=26), and non-pregnant mothers (n=69). | Children had a significant effect on women’s sleep-wake cycle and chronotype. Women with children, older women and pregnant women were more likely to be earlier chronotypes.  Women with children had the lowest social jetlag (difference between weekends and weekday).  Synchrony between partners increased in pregnancy and returned to pre-birth levels after the birth of the child. Synchrony between the mother and child was stronger than synchrony between the mother and partner.  Chronotypes were correlated between mothers, partners and their children.  Partners chronotypes were highly correlated and there was a correlation between mother and child, but not between partner and child. |
|  | Relationship Between Child Sleep Disturbances and Maternal Sleep,  Mood, and Parenting Stress: A Pilot Study  Meltzer and Mindell  2007 (Meltzer and Mindell 2007) | Pilot study. | Pearson’s correlation.  Multivariate analysis of variance. | No. | There were no significant correlations between parent and child sleep variables (bedtime, wake time and total sleep time), but significant relationships were found between maternal sleep quality and sleep onset latency in children, and maternal sleep quality and child sleep disruptions. |
|  | Family synchronizers: Predictors of sleep–wake rhythm for Japanese first-time mothers.  Yamazaki  2007 (Yamazaki 2007) | Longitudinal. | A 4-stage hierarchical multiple regression analysis. | No. | Most infant variables were not significantly correlated with the mother’s rhythm strength.  The three significant predictors for first time mothers sleep wake rhythm strength during the early postpartum period were household income, chronotype during pregnancy and the father’s daily social rhythm during the early postpartum period.  Mothers with evening chronotype had stronger circadian rhythms post-partum. |
|  | Infant sleep and feeding pattern: Effects on maternal sleep  Thomas et al.  2005 (Thomas and Foreman 2005) | Cross-sectional. | Regression and post hoc analysis. | No. | Maternal sleep is driven by infant sleep-wake and feeding patterns.  Increased time feeding decreased infant and maternal sleep.  The length of the infants’ longest sleep period was directly related to duration of maternal longest sleep period and indirectly related to number of maternal sleep episodes.  Infants with higher weight gain had more total sleep and longer sleep duration. Infant’s gender affected sleep and feeding patterns.  Maternal age affects maternal and infant sleep. |
|  | Similarities and Differences in Sleep-Wake Patterns Among Adults and Their Children  Gau and Merikangas.  2004 (Gau and Merikangas 2004) | Cross-sectional study. | Pearson and Spearman correlation and analysis of variance using a mixed method model. | No. | Correlations between parents and children were low in terms of morning/eveningness score, frequency of daytime napping, sleep needed to maintain daytime functioning, bedtime, wake time, duration of sleep during weekdays and bedtime and rise times on weekends.  There is a significant difference in parent-child sleep variables across school grade levels. |
|  | The development of infants’ circadian rest–activity rhythm and mothers’ rhythm  Nishihara et al.  2002 (Nishihara et al. 2002) | Longitudinal. | Autocorrelograms.  . | No. | Infant’s sleep-wake rhythm had already begun in the 3^rd^ week.  There was a 24-hour peak for infants in the 3^rd^ week. The amplitude of this peak was the smallest of all the weeks and the regularity of circadian rhythm was the weakest. From the 6^th^ to the 12^th^ week the amplitude of the circadian rhythm gradually increased.  The amplitude of the 24-hour peak of the mother’s circadian rhythm at the 3^rd^ week was the smallest of all weeks and it increased from the 6^th^ to 12^th^ week. This was influenced by the mother’s movements when taking care of her infant at night.  There was strong synchronisation of mother’s wakefulness and infant’s movement at night after postpartum weeks 1-6.  Authors conclude that mother-infant synchronisation is probably the first factor in the entrainment of the infant’s sleep-wake rhythm. |
|  | Mothers’ wakefulness at night in the post-partum period  is related to their infants’ circadian sleep–wake rhythm.  Nisihara.  2000 (Nishihara et al. 2000) | Longitudinal | Autocorrelation.  ANOVAs.  Pairwise comparisons. | No. | The mothers’ night-time movements significantly decreased from Week 3 to Week 12. This was related to their infants developing a circadian sleep-wake rhythm.  Eighty per cent of infants showed a prominent circadian component between Weeks 8 and 11. All infants showed a 24-hour peak at week 12. |
|  | Activity Monitoring of the Inhabitants in Tauwema, a Traditional Melanesian Village:  Rest/Activity Behaviour of Trobriand Islanders (Papua New Guinea)  Siegmund et al.  1998 (Siegmund et al. 1998) | Cross-sectional. | Visual comparison.  Inter-individual comparisons.  Phase correlations. | No. | Circadian rhythms emerge from ultradian patterns in the first few months of life.  Rhythmicity of adults was related to the light-dark cycle with a strong social component.  The mean sleep of younger infants was 9-12 hours per day and 7-10 hours per day for adults.  On average, wives slept longer than their husbands. |
|  | Sleep and arousal, synchrony and independence, among mothers and infants sleeping apart and together (same bed): an experiment in evolutionary medicine  McKenna and Mosko (McKenna and Mosko 1994)    1994 | Two pilot studies. | Descriptive. | No. | Co-sleeping infants arise more frequently and with greater overlap with mother arousals. This implies sleep arousals are partner induced.  Infant sleep stages are altered by co-sleeping – decreased sleep stages 3 and 4 and greater simultaneous overlap with mother sleep-wake cycles.  Co-sleeping mothers and infants spend more time in the same sleep stage or awake condition. |
